# PRDM1 Drives a TIM3+ Macrophage Immunosuppressive Niche via LGALS9 Signaling in Prostate Cancer Progression

**DOI:** 10.32604/or.2026.079316

**Published:** 2026-06-16

**Authors:** Yangyang Zhang, Ilyar Mamtili, Yonghao Chen, Guangjie Ji, Chaozhao Liang

**Affiliations:** 1 Department of Urology, The First Affiliated Hospital of Anhui Medical University, Hefei, China; 2 Institute of Urology, Anhui Medical University, Hefei, China; 3 Anhui Province Key Laboratory of Genitourinary Diseases, Anhui Medical University, Hefei, China; 4 Department of Anesthesiology, Shanghai Xuhui Central Hospital, Shanghai, China; 5 West China Medical Center, Sichuan University, Chengdu, China

**Keywords:** Prostate cancer, single-cell, macrophage, immunoregulator, disease subtyping

## Abstract

**Background:** Prostate cancer (PCa) responds poorly to immunotherapy. We investigated the myeloid checkpoint TIM3 (HAVCR2) to define its lineage localization and regulatory logic in the PCa microenvironment. **Methods:** We integrated stage-resolved public single-cell RNA-seq datasets spanning primary PCa, metastatic hormone-sensitive PCa, and castration-resistant PCa. Myeloid compartments were analyzed via differential expression, regulon inference, and ligand–receptor modeling. Clinical relevance was evaluated in the Cancer Genome Atlas prostate adenocarcinoma (TCGA-PRAD) cohort and independent cohorts using a myeloid TIM3 signature. Mechanistic validation was achieved through PR domain zinc finger protein 1 (PRDM1) chromatin immunoprecipitation followed by Chromatin Immunoprecipitation (ChIP)–qPCR (ChIP-qPCR), TIM3-promoter luciferase assays, and functional perturbation of the galectin 9 (LGALS9)-TIM3 signaling pathway in macrophages differentiated. **Results:** TIM3 expression was predominantly confined to monocytes/macrophages, indicating TIM3 as a microenvironmental checkpoint in PCa. TIM3_high macrophages formed a Secreted Phosphoprotein 1 (SPP1)-enriched tumor-associated macrophage (TAM) state coupled to chemokine programs and extracellular matrix remodeling. Regulon profiling nominated PR domain zinc finger protein 1 (PRDM1) as an upstream driver; PRDM1 correlated with TIM3. Communication inference further highlighted an LGALS9-TIM3 axis and a C-X-C motif chemokine receptor 4/integrin subunit beta 1 (CXCR4/ITGB1)-associated permissive niche. Recombinant LGALS9 induced TIM3-linked M2-like macrophages polarization and increased CXCR4/ITGB1, which was attenuated by TIM3 blockade. **Conclusions:** Our results delineate a PRDM1-licensed TIM3_high macrophage program sustained by an LGALS9-TIM3 reinforcement loop and coupled to immunosuppressive and remodeling-associated phenotypes. Targeting TIM3 in the myeloid compartment, alone or in rational combinations, may represent a feasible strategy to reprogram tumor-associated macrophage states in PCa.

## 1 Introduction

Prostate cancer (PCa) remains a major contributor to cancer-related morbidity and mortality among men worldwide, and disease progression is frequently driven by therapeutic resistance in metastatic and castration-resistant settings [1,2,3]. While immune checkpoint inhibitors have reshaped standard-of-care treatment across multiple malignancies, clinical activity in unselected PCa has been modest, with benefit largely confined to molecularly defined subsets [4,5,6]. These observations underscore the need to delineate microenvironmental mechanisms that constrain antitumor immunity and to identify actionable, state-specific checkpoints that may enable rational combination strategies.

A dominant feature of the PCa tumor microenvironment is its substantial myeloid compartment, including tumor-associated macrophages (TAMs), which can promote tumor growth through immune suppression, cytokine and chemokine networks, angiogenesis, and extracellular matrix (ECM) remodeling [5,6,7]. However, TAM biology in PCa is heterogeneous and context-dependent, and bulk transcriptomic analyses often obscure the cellular sources and state-specific programs that underpin clinically relevant immunoregulatory phenotypes. Thus, cell-resolution mapping across disease stages is essential to distinguish which immune subsets express actionable checkpoints, how those checkpoints align with functional programs, and which upstream regulators or extrinsic ligands sustain these states [8,9,10].

TIM3 (encoded by HAVCR2) is an emerging checkpoint receptor implicated in both lymphoid and myeloid regulation [11,12]. While TIM3 has been studied in T-cell exhaustion and in myeloid suppressive programs in other malignancies, the cellular localization of TIM3 in PCa, its association with specific TAM states, and its transcriptional and microenvironmental regulatory logic remain insufficiently defined [13,14]. In particular, whether TIM3 reflects a stable macrophage state governed by upstream transcription factors, and whether ligand-driven reinforcement contributes to persistence of TIM3–high niches, are key open questions with direct translational implications [15].

This study aimed to characterize the molecular features, functional role, and clinical relevance of TIM3-high TAM in PCat. We integrated stage-resolved single-cell RNA sequencing datasets spanning primary PCa, metastatic hormone-sensitive disease, and castration-resistant disease to map HAVCR2 expression at single-cell resolution and identify TIM3-associated TAM programs. We further combined single-cell and spatial multi-omics data, bulk transcriptomic cohorts, and targeted experimental validation to clarify the biological and clinical significance of TIM3-positive macrophages in PCa.

## 2 Materials and Methods

### 2.1 Data Sources and Pre-Processing

We collected and integrated multi-omics data from publicly available repositories to characterize the transcriptomic landscape of prostate cancer. Single-cell RNA sequencing data were obtained from the Gene Expression Omnibus (GSE274229, https://www.ncbi.nlm.nih.gov/geo), which included 44 human tumor tissue samples, including 13 primary prostate cancer, 25 mHSPC, and 6 CRPC, and was used to identify distinct cellular subpopulations and functional states within the tumor microenvironment. To test the results from GSE274229, we enrolled the independent single-cell RNA-seq dataset (GSA accession: HRA000823, https://ngdc.cncb.ac.cn/gsa-human/), which included ten PCa samples.

Spatial transcriptomics data were derived from the 10× Genomics website (https://www.10xgenomics.com/cn), providing spatially resolved information on cellular localization and tissue microenvironmental organization. The analyzed section corresponded to B1 Block 1D1061-Tp11, Section 1 (slide V11J26-002, area B1). For bulk transcriptomic analyses and cohort-level validation, RNA-seq expression profiles and corresponding clinical annotations were retrieved from TCGA-PRAD, the GEO cohort GSE116918, and an independent prostate cancer cohort from the International Cancer Genome Consortium (ICGC; PRAD-CA).

### 2.2 Single-Cell Transcriptomic Data Processing

The single-cell transcriptomic dataset GSE274229 (N = 44) was processed using the Seurat framework with a rigorous quality control pipeline [16]. Low-quality cells were excluded by filtering out those with low unique molecular identifier (UMI) counts and those exhibiting an excessive proportion of mitochondrial transcripts, thereby minimizing technical artifacts. To capture meaningful transcriptional variation across cells, we applied the variance-stabilizing transformation method and selected the top 3000 highly variable genes, which provided the basis for subsequent cell state identification. Dimensionality reduction was performed using principal component analysis, and potential batch effects were corrected with the Harmony algorithm to ensure consistent integration of cells originating from different tumor samples [17]. A shared nearest neighbor graph was then constructed, followed by multi-resolution clustering with resolution parameters ranging from 0.1 to 1.0 in increments of 0.2, which allowed the detection of distinct cellular subpopulations within the tumor microenvironment. We selected the best number of principal components based on the elbow plot and the variance explained by each component. For visualization, Uniform Manifold Approximation and Projection (UMAP) was performed to visualize the data based on the top 15 principal components. Annotation of clusters was guided by canonical cell type-specific markers and further refined with automated tools such as GPTCelltype [18], resulting in accurate classification and labeling of major and minor cellular subpopulations. Curated single-cell subpopulations were subsequently integrated with spatial transcriptomics data and bulk sequencing cohorts to define candidate features, which were further incorporated into downstream machine learning pipelines for prognostic modeling.

### 2.3 Spatial Transcriptomic Data Processing

Spatial transcriptomic data from the 10× Genomics platform (https://www.10xgenomics.com/cn) were processed using a combination of Seurat and SpatialExperiment workflows. Quality control was first applied to remove low-quality spots. Spots with a mitochondrial gene percentage >20% or total UMI counts <500 were excluded from downstream analysis. Normalization of the raw expression matrices was performed with SCTransform, which effectively accounted for variability in sequencing depth across tissue sections. Principal component analysis was then conducted for dimensionality reduction, and clustering based on the top 20 principal components allowed the identification of spatially coherent regions with distinct transcriptional profiles. To directly link molecular signatures with tissue architecture, functions such as SpatialFeaturePlot and SpatialDimPlot were used to overlay gene expression patterns and cell cluster assignments onto the corresponding histological images.

### 2.4 Myeloid Subclustering and TAM State Annotation

The myeloid compartment was reprocessed using SCTransform, dimensionality reduction, and graph-based clustering as above (resolution = 0.5), to resolve TAM and related myeloid states. Subclusters were annotated based on state markers (SPP1, APOE, C1QC, FOLR2, CXCL9, S100A8, CD1E) and cross-validated using published macrophage state signatures where appropriate.

### 2.5 Definition of TIM3_High and TIM3_Low Groups

To derive an objective TIM3 stratification aligned to clinical endpoints, we computed myeloid pseudo-bulk HAVCR2 per sample. Samples were dichotomized into TIM3_high and TIM3_low groups based on the median values. The resulting groups were used for state composition analyses, differential expression, regulon inference, and communication modeling.

### 2.6 Differential Expression and Pathway Enrichment

Differential expression between TIM3_high and TIM3_low myeloid cells was performed using Wilcoxon rank-sum tests with multiple testing correction (Benjamini–Hochberg). Genes with FDR < 0.05 and |log_2_ Fold Change (FC)| > 0.25 were considered significant. Functional enrichment was conducted using Gene Ontology and pathway analyses based on the clusterProfiler (https://github.com/YuLab-SMU/clusterProfiler) package [19,20,21,22], and gene set enrichment analysis (GSEA) was performed using ranked statistics with FDR control.

### 2.7 Transcription Factor Regulon Inference and Activity Scoring

Gene regulatory networks and transcription factor (TF) activities were inferred from single-cell RNA-seq data using the SCENIC (v. 0.12.1) framework as implemented in OmicVerse (v. 1.7.6) [23]. Briefly, candidate TF-target relationships were first inferred from gene expression profiles (raw count matrix) using a co-expression–based network inference step with tree-based ensemble regression, generating an initial TF-target adjacency network. Specifically, candidate TF-target relationships were inferred using GENIE3 with 1, 000 trees. Next, motif enrichment and cis-regulatory pruning were applied to refine the network and retain putative direct TF targets, thereby defining high-confidence regulons. The cisTarget human motif database was retrieved from https://resources.aertslab.org/cistarget/databases/homo_sapiens/hg38/refseq_r80/. Regulon activity was subsequently quantified at single-cell resolution using AUCell, which computes an AUC-based enrichment score of each regulon’s gene set within the ranked expression profile of each cell, yielding a regulon activity matrix for downstream analyses.

### 2.8 Cell-Cell Communication Analysis

Intercellular communication was inferred with CellChat using its built-in curated ligand–receptor interaction database. Rather than reconstructing ecosystem-wide networks by condition, we focused on TIM3-stratified macrophage states and quantified communication between TIM3_high or TIM3_low macrophages and all other annotated cell populations. Specifically, macrophages were partitioned into TIM3_high and TIM3_low subsets based on the median expression of HAVCR2, and CellChat was applied to estimate communication probabilities for ligand-receptor pairs connecting each macrophage subset with other cell types using the default parameter implemented in the CellChat workflow.

### 2.9 Multiplex Immunofluorescence Analysis

Multiplex immunofluorescence (mIF) staining was conducted on formalin-fixed, paraffin-embedded (FFPE) tissue sections (4 μm thick) following a sequential protocol. FFPE specimens were obtained from 10 patients who underwent radical prostatectomy at the Department of Urology, The First Affiliated Hospital of Anhui Medical University. The study protocol was approved by the Ethics Committee of The First Affiliated Hospital of Anhui Medical University (Ethics Approval No.: 2026-1-01-76), and all procedures involving human participants were conducted in accordance with the Declaration of Helsinki. Written informed consent was obtained from all patients prior to sample collection. The basic information of the relevant patients is provided in Supplementary Table S1. Briefly, after deparaffinization and rehydration, antigen retrieval was performed under heat in a decloaking chamber using either EDTA buffer (pH 9.0) or citrate buffer (pH 6.0), with the specific buffer and condition optimized for each target antigen. Following quenching of endogenous peroxidases with 3% hydrogen peroxide (25 min, room temperature), sections were blocked using 3% bovine serum albumin (BSA) (Solarbio, Beijing, China, Cat# A8020) for 10 min. Prior to each staining cycle, sections were incubated with a specific primary antibody (diluted in blocking buffer) overnight at 4°C. After washing with PBS, a corresponding horseradish peroxidase (HRP)-conjugated secondary antibody (Proteintech, Cat: RGAR011, 1:1000, Wuhan, China) was applied and incubated for 1 h at room temperature. Target antigens were visualized using a tyramide signal amplification (TSA) system with a fluorophore-conjugated tyramine reagent. Following image acquisition or signal inactivation, the complex of primary antibody, secondary antibody, and fluorophore was thoroughly stripped via another round of heat-induced epitope retrieval. This process—comprising sequential incubation with primary antibody, HRP-secondary antibody, fluorophore-tyramine development, and signal removal—was repeated iteratively to label multiple antigens on the same tissue section. After all target antigens were labeled, cell nuclei were counterstained with DAPI (HuiLan Biotech, Cat: RC05, Shanghai, China) for 10 min.

Finally, slides were scanned using a multispectral or multichannel whole-slide fluorescence scanner for subsequent image analysis. The following primary antibodies were employed: anti-TIM3 (Cat# 45208T, 1:200, Cell Signaling Technology, Danvers, MA, USA), anti-LGALS9 (Cat# 40947, 1:200, SAB, College Park, MD, USA), anti-PD-L1 (Cat# CY5980, 1:200, Abways, Shanghai, China), anti-CD163 (Cat# ab182422, 1:1500, Abcam, Cambridge, UK), anti-TGFB (Cat# ab215715, 1:500, Abcam), anti-FN1 (Cat# ab2413, 1:200, Abcam).

### 2.10 Chromatin Immunoprecipitation (ChIP)–qPCR

ChIP–qPCR was performed to assess PRDM1 occupancy at the TIM3 promoter in PC-3 prostate cancer cells (ATCC, CRL-1435, Manassas, VA, USA). PC-3 cells were cultured in RPMI-1640 medium (Gibco, Cat# 11875093 Grand Island, NY, USA) supplemented with 10% fetal bovine serum (FBS, Gibco Cat# A5256701) at 37°C in a humidified atmosphere containing 5% CO_2_. Cells were routinely tested and confirmed to be free of mycoplasma contamination, and cell line identity was verified by short tandem repeat (STR) profiling. Cells were cross-linked with 1% formaldehyde for 10 min at room temperature, quenched with glycine, and lysed to isolate chromatin. Chromatin was sonicated to fragments of approximately 200–500 bp and immunoprecipitated overnight at 4°C using anti-PRDM1 antibody (Cat# 14-5963-82, Invitrogen/eBioscience, Thermo Fisher Scientific, Waltham, MA, USA; clone 6D3; 5 μg per reaction) or matched rat IgG2a kappa isotype control (Cat# 14-4321-82, Invitrogen/eBioscience; 5 μg per reaction). After reverse crosslinking and DNA purification, enrichment at the predicted PRDM1-bound region within the HAVCR2 promoter was quantified by qPCR using the following primers: forward, 5′-AGAGGCTTTGGCCATGAATG-3′; reverse, 5′-AAGGTCACACTCCCAGAGC-3′. qPCR was performed on an Applied Biosystems QuantStudio 7 Real-Time PCR System (Applied Biosystems, Thermo Fisher Scientific) using PowerUp SYBR Green Master Mix (Applied Biosystems, Thermo Fisher Scientific; Cat# A25742) under the following cycling conditions: 95°C for 5 min, followed by 40 cycles of 95°C for 10 s and 60°C for 30 s, with melt-curve analysis performed at the end of amplification. ChIP-qPCR experiments were performed with three independent biological replicates, and each sample was analyzed in triplicate as technical replicates.

### 2.11 Luciferase Reporter Assay

To investigate whether PRDM1 directly transactivates the HAVCR2 promoter, a dual-luciferase reporter assay was performed in HEK293T cells (ATCC, CRL-3216, Manassas, VA, USA), whose identity was verified by short tandem repeat (STR) profiling and which were routinely tested and confirmed to be free of mycoplasma contamination before use. Cells were seeded into 24-well plates at 9 × 10^4^ cells per well in 500 μL complete medium 24 h before transfection, reaching approximately 80% confluence on the day of transfection. The wild-type human HAVCR2 promoter fragment was cloned into the pGL3-Basic firefly luciferase reporter vector (Promega, Cat# E1751, Madison, WI, USA). A mutant HAVCR2 promoter reporter was generated by site-directed mutagenesis of the predicted PRDM1-binding core motif, in which the sequence 5′-GAAAG-3′ was mutated to 5′-TCCCT-3′ to disrupt PRDM1 binding. The PRDM1 overexpression plasmid used in this study was a human PRDM1 expression clone (OriGene, Cat# RG222766, Rockville, MD, USA). The Renilla luciferase internal control plasmid used for normalization was the pRL-TK Vector (Promega, Cat# E2241, Madison, WI, USA). For each well of a 24-well plate, cells were co-transfected with 400 ng of HAVCR2 promoter firefly reporter plasmid (wild-type or mutant), 80 ng of PRDM1 expression plasmid or the corresponding empty vector, and 20 ng of pRL-TK Renilla plasmid, with the total DNA amount adjusted to 500 ng per well. Transfection was performed using Lipofectamine 3000 Transfection Reagent (Invitrogen, Cat# L3000015, Carlsbad, CA, USA) together with P3000 Reagent, according to the manufacturer’s protocol for 24-well plates. Transfection variability across conditions was controlled by maintaining identical cell density, DNA amount, and reagent ratios in all groups, and firefly luciferase activity was normalized to Renilla luciferase activity. Cells were harvested 48 h after transfection, and luciferase activity was measured using the Dual-Luciferase Reporter Assay System (Promega, Cat# E1910, Madison, WI, USA). Firefly luciferase activity was normalized to Renilla luciferase activity, and the results were presented relative to the corresponding control group. All experiments were independently repeated three times, and each condition was analyzed in technical triplicate.

### 2.12 Reverse Transcription Quantitative PCR (RT-qPCR)

The THP-1 cell line (CAT: SNL-044, Sunncell, Wuhan, China) was cultured as human macrophages in RPMI 1640 medium (Sigma Aldrich, Cat#D5796, St. Louis, MO, USA) supplemented with 10% FBS (KeyGEN Biotech, Cat: KGA6006-50, Nanjing, China) and 1% penicillin/streptomycin (Procell, Cat: PB180120, Wuhan, China). Cell line identity was confirmed by short tandem repeat (STR) profiling and confirmed to be mycoplasma-free before the experiments. THP-1 monocytes were seeded in 96 or 12-well plates at densities of 8 × 10^4^ or 8 × 10^6^ cells per well, respectively, and supplemented with 50 ng/mL phorbol 12-cassia bark glycolide 13-acetate (PMA, Sigma Aldrich, Cat: P1585-5MG) for 24 h to induce differentiation. Cells were treated as indicated (PBS, LGALS9, or LGALS9 + anti-TIM3) prior to RNA extraction. Total RNA was extracted using TRIzol reagent (Invitrogen, Cat#15596026, MA, USA) according to the manufacturer’s protocol. Reverse transcription was performed using PrimeScript™ RT reagent Kit (Takara, Cat#RR047Q, Beijing, China) in a total reaction volume of 20 μL. The reverse transcription conditions were as follows: 25°C for 5 min, 42°C for 30 min, 85°C for 5 min.qPCR was performed using PowerTrack™SYBR Green Master Mix (Invitrogen, Cat#A46109, MA, USA) in a total volume of 20 μL per reaction on a Biometra Tone 96G (AnalytikJena, Jena, Germany). The thermal cycling conditions were: initial denaturation at 95°C for 3 min, followed by 40 cycles of 55°C for 20 s and 72°C for 20 s, with a final melt curve analysis. Gene expression levels of TIM3, LGALS9, CD206, ARG1, CXCR4, ITGB1, and IL10 were quantified. GAPDH was used as an internal control. Relative gene expression was calculated using the 2^−^^ΔΔCt^ method and normalized to the Control group. All RT-qPCR experiments were performed with three independent biological replicates, and each sample was analyzed in three technical replicates. Messages for all primers are available in the Supplementary Table S2.

### 2.13 Western Blot Analysis

Following differentiation, THP-1-derived macrophages were allocated into three groups: PBS control, LGALS9, and LGALS9 + anti-TIM3. For stimulation, cells were treated with 10 μg/mL recombinant human LGALS9 (R&D Systems, Cat# 2045-GA/CF, Minneapolis, MN, USA) for 24 h. In the neutralization assay, a functional blocking anti-TIM3 antibody (10 μg/mL; Invitrogen/eBioscience, Cat# 16-3109-85, clone F38-2E2) was co-administered.

Total protein was extracted using RIPA buffer (Thermo Fisher Scientific, Waltham, MA, USA; Cat# 89900) supplemented with Protease/Phosphatase Inhibitor Cocktails (Selleck Chemicals, Houston, TX, USA; Cat# K4000/K5000) and quantified via BCA assay (Thermo Fisher Scientific; Cat# 23225). Briefly, 20 μg of protein per sample was resolved by 10% SDS-PAGE, transferred onto PVDF membranes (Thermo Fisher Scientific; Cat# 88518), and blocked with 5% non-fat milk for 1 h at room temperature.

Membranes were probed overnight at 4°C with primary antibodies against: TIM3 (Cell Signaling Technology, Danvers, MA, USA; Cat# 45208), ARG1 (Abcam; Cat# ab133543), CXCR4 (Abcam; Cat# ab124824), CD206 (Abcam; Cat# ab125028), ITGB1 (Abcam; Cat# ab179471), and GAPDH (Abcam; Cat# ab9484). After washing, blots were incubated with HRP-conjugated secondary antibodies (Proteintech, Wuhan, China; Goat Anti-Rabbit, Cat# SA00001-2; or Goat Anti-Mouse, Cat# SA00001-1; 1:5000) for 1 h at room temperature. Signals were visualized using Pierce ECL substrate (Thermo Fisher Scientific, USA; Cat# 32106), with GAPDH as the loading control. All experiments were performed in three independent biological replicates.

### 2.14 Flow Cytometry Analysis

Flow cytometry was performed to analyze macrophage phenotypes. Cells from each treatment group (PBS, LGALS9, and LGALS9 + anti-TIM3) were harvested, washed, and blocked with Fc receptor blocking solution (Invitrogen/eBioscience, clone F38-2E2, Cat# 16-3109-85), followed by staining with fluorochrome-conjugated antibodies against TIM3 (BioLegend, San Diego, CA, USA; clone F38-2E2, Cat# 345006), CD206 (BioLegend, San Diego, CA, USA; clone 15-2, Cat# 321109), CD163 (BioLegend, San Diego, CA, USA; clone GHI/61, Cat# 333617), and CXCR4 (BioLegend, San Diego, CA, USA; clone 12G5, Cat# 306514). Corresponding isotype controls were included to define positive gates. Cells were analyzed on a flow cytometer (Beckman Coulter CytoFLEX LX, Beckman Coulter, Brea, CA, USA), and data were processed using FlowJo software (FlowJo10.8, BD Biosciences, Ashland, OR, USA). For data acquisition, we recorded a minimum of 20,000 events per sample to ensure sufficient cell population coverage. To maintain experimental rigor, all flow cytometry assays were independently replicated across three separate biological batches, with each specific treatment group analyzed in triplicate as technical replicates.

### 2.15 Statistical Analysis

All tests were two-sided. Continuous variables were compared using the Wilcoxon rank-sum test (two groups) or Kruskal–Wallis test (multiple groups), and categorical variables using *χ*^2^ or Fisher’s exact test as appropriate. Multiple comparisons were adjusted by the Benjamini–Hochberg method. Survival was analyzed with the log-rank test, and hazard ratios were estimated using Cox proportional hazards models with proportionality assessed by Schoenfeld residuals. Analyses were performed in R v4.2.3 and Python v3.7. A two-tailed *p* < 0.05 was considered significant.

## 3 Results

### 3.1 Lineage-Resolved Single-Cell Profiling Confines TIM3 to Myeloid Cells in Prostate Cancer

**Figure 1:**
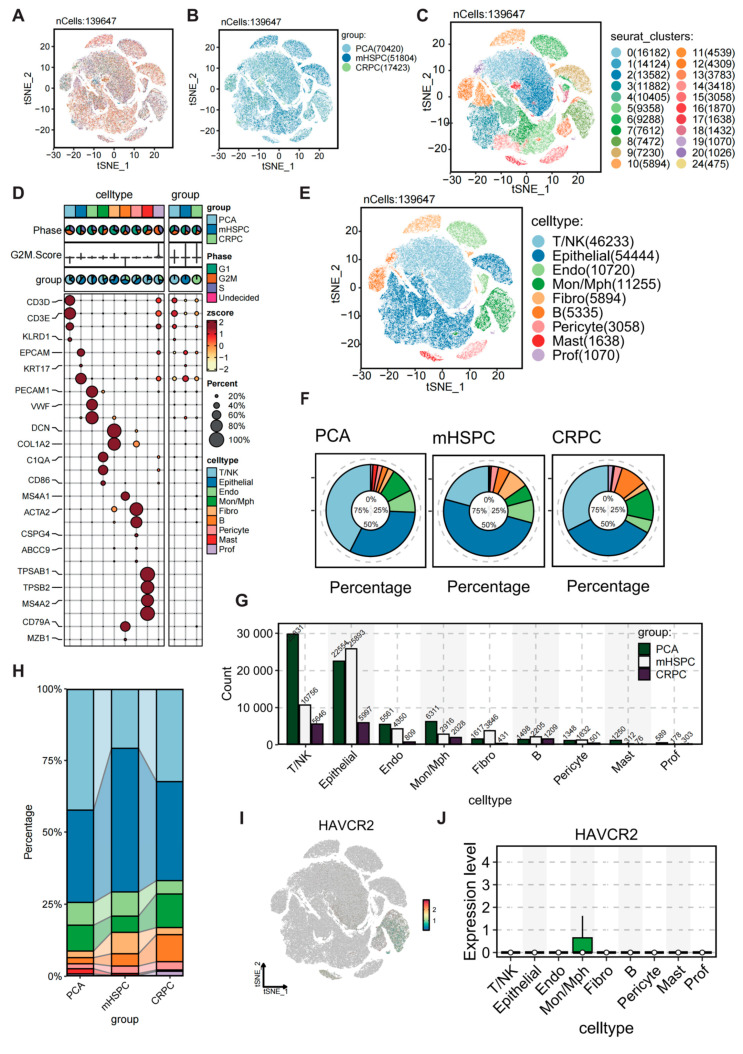
**Lineage-resolved single-cell profiling confines HAVCR2/TIM3 to myeloid cells in prostate cancer (PC).** (**A**) UMAP of the integrated single-cell dataset colored by samples (n = 139,647). (**B**) UMAP colored by groups: PCA (n = 70,420), mHSPC (n = 51,804), and CRPC (n = 17,423). (**C**) UMAP colored by Seurat clusters (**D**) UMAP colored by major cell types. (**E**) Dot plot of representative marker genes and cell-cycle metrics (Phase and G2M.Score) supporting cell-type annotation. Dot size indicates the fraction of expressing cells and color denotes scaled average expression (z-score). (**F**) Donut plots showing cell-type composition within each clinical group. (**G**) Stacked bar plot comparing cell-type proportions across groups. (**H**) Bar plot showing absolute cell counts of each cell type stratified by group. (**I**) Feature plot of HAVCR2 expression projected onto the UMAP embedding. (**J**) Distribution of HAVCR2 expression across annotated cell types.

We assembled an integrated single-cell atlas spanning primary prostate cancer (PCa), metastatic hormone-sensitive disease (mHSPC), and castration-resistant prostate cancer (CRPC) (Fig. 1A,B). After quality control and batch-aware integration, the atlas comprised 139,647 cells (PCA 70,420; mHSPC 51,804; CRPC 17,423) and resolved the major cellular lineages by canonical markers, including epithelial cells (54,444), T/NK cells (46,233), endothelial cells (10,720), monocytes/macrophages (Mon/Mph) (11,255), fibroblasts (5894), B cells (5335), pericytes (3058), mast cells (1638), and proliferating cells (1070) (Fig. 1C–E). Notably, marked differences in cellular composition were observed across the three disease stages, with distinct shifts in the proportions of major cell lineages between primary PCa, mHSPC, and CRPC (Fig. 1F–H). TIM3 is increasingly recognized as a checkpoint in addition to its role in exhausted T cells, and mechanistic work has shown that TIM3 can directly promote a tumor-supportive, M2-like macrophage program through intracellular signaling rewiring [24]. Consistently, recent cross-tumor studies have defined conserved TIM3^+^ TAM niches associated with resistance to immunotherapy, providing a strong translational rationale to resolve the cellular localization and state specificity of TIM3 in prostate cancer [14]. Mapping TIM3 onto the unified embedding revealed a strikingly lineage-constrained expression pattern: HAVCR2 expression was relatively lower in epithelial and most stromal compartments, and was comparatively enriched within the Mon/Mph lineage, where it represented the predominant source of transcript signal across the atlas (Fig. 1I,J) To further validate our findings, we analyzed an independent single-cell RNA-seq cohort. Major cell types were annotated using canonical marker genes (Fig. S1A,B), and consistent with our primary analysis, HAVCR2 expression was relatively enriched in the macrophage compartment compared with other cell lineages (Fig. S1C,D).

TIM3 has been widely recognized as a checkpoint associated with dysfunctional tumor-infiltrating T cells and often acts synergistically with PD-1-related T cell depletion procedures [25]. Notably, accumulating evidence indicates that TIM3 is also prominently expressed and functionally active in intratumoral myeloid populations, where it constrains innate sensing and chemokine outputs that are critical for productive antitumor immunity [26]. This segmentation designates myeloid cells as the primary cellular environment for TIM3 biology in prostate cancer, prompting subsequent analyses to explore the state of TIM3-associated macrophages and their microenvironmental interactions.

### 3.2 Myeloid Re-Clustering Exposes Discrete TAM Circuits and a Stage-Remodeled TIM3–Positive Niche

**Figure 2:**
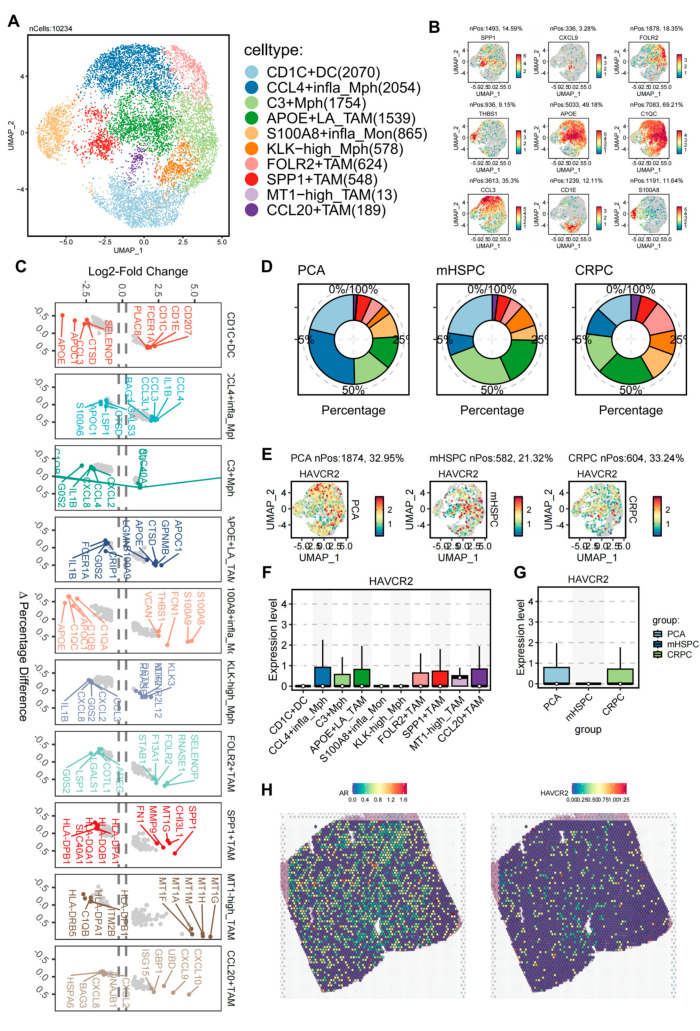
Myeloid subclustering identifies Tumor-associated macrophage (TAM) heterogeneity and stage-resolved TIM3 (HAVCR2) positivity. (**A**) UMAP of re-clustered myeloid cells (n = 10,234) identifying major subsets including CD1C^+^ dendritic cells, inflammatory monocytes/macrophages, and multiple TAM states (cell numbers per subset are indicated). (**B**) Feature plots of representative markers (SPP1, THBS1, FOLR2, CXCL9, APOE, C1QC, CCL3, CD1E, S100A8) supporting subset annotation. (**C**) Marker summary for each myeloid subset. Scatter plots depict differential expression metrics relative to other myeloid cells. (**D**) Donut plots showing the relative composition of myeloid subsets across clinical groups prostate cancer (PCA), metastatic hormone-sensitive prostate cancer (mHSPC), castration-resistant prostate cancer (CRPC). (**E**) Stage-stratified HAVCR2 feature plots with the number and percentage of HAVCR2-positive cells annotated for each group. (**F**) HAVCR2 expression across myeloid subsets (boxplots). (**G**) HAVCR2 expression across clinical groups (boxplots). (**H**) Spatial transcriptomics maps showing AR and HAVCR2 expression across tissue spots.

Within the integrated prostate cancer single-cell atlas, we re-clustered myeloid cells (n = 10,234) and resolved a structured spectrum encompassing dendritic cells, inflammatory monocytes/macrophages, and multiple TAM states, including CCL4^+^ inflammatory macrophages, C3^+^ macrophages, APOE^+^ lipid-associated TAMs, SPP1^+^ TAMs, FOLR2^+^ TAMs, and CCL20^+^ TAMs (Fig. 2A). These subsets displayed concordant, spatially segregated marker patterns across the UMAP embedding—such as SPP1, THBS1, FOLR2, CXCL9, APOE, C1QC, CCL3, CD1E, and S100A8—and were further delineated by differential-expression effect sizes and differences in the fraction of expressing cells, collectively supporting robust annotation and pronounced functional heterogeneity (Fig. 2B,C). Comparisons across disease stages revealed marked shifts in myeloid subset composition among PCA, mHSPC, and CRPC, consistent with systemic remodeling of the myeloid niche during progression. Among them, APOE, SPP1+Mph and other cells were significantly increased in mHSPC and CRPC, which was consistent with the previously reported results (Fig. 2D) [5]. Against this cellular framework, TIM3 exhibited clear stage-associated variation in both expression level and positivity rate, and, at the state level, preferentially localized to specific TAM-like subsets rather than being uniformly expressed across all myeloid cells, indicating that TIM3 marks a selective myeloid program (Fig. 2E–G). Spatial transcriptomics analysis revealed that HAVCR2 signaling was confined to focal, patchy hotspots, which is distinct from the broader AR distribution. This provides orthogonal evidence that TIM3 reflects a compartmentalized, microenvironment-dependent macrophage state rather than a discrete cell population, prompting us to further investigate cell communication and ligand-receptor enhancement mechanisms (Fig. 2H).

### 3.3 TIM3_High Myeloid Cells Couple HAVCR2 Elevation to an SPP1^+^ TAM State and a Remodeling-Centric Transcriptional Program

**Figure 3:**
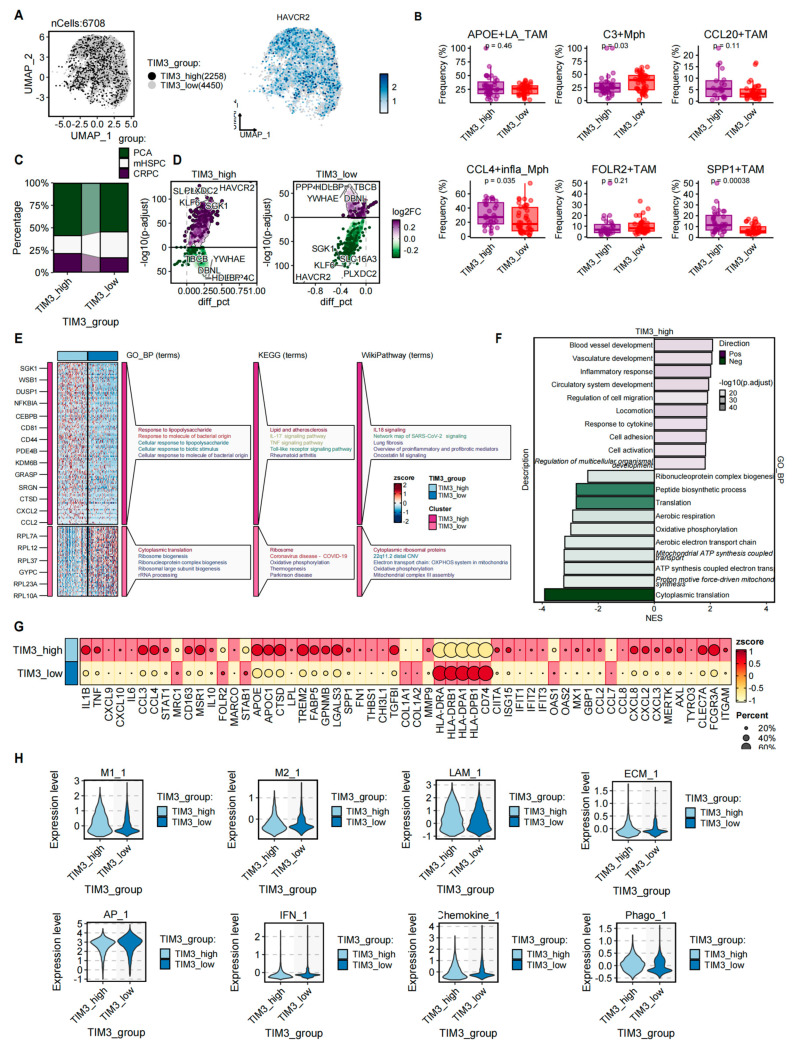
TIM3_high myeloid cells couple HAVCR2 elevation to an SPP1^+^ TAM state and a remodeling-centric transcriptional program. (**A**) UMAP showing TIM3_high/low assignment and HAVCR2 expression (n_high = 2258; n_low = 4450). (**B**) Subcluster frequency comparison highlighting SPP1^+^ TAM enrichment (*p* = 3.8 × 10^−^^4^). (**C**) Distribution of TIM3_high/low across disease groups and/or subclusters. (**D**) Differential expression summary (TIM3_high vs. TIM3_low). (**E**) Heatmap of representative differentially expressed genes (z-score scaled) and functional enrichment of group-specific gene sets across GO Biological Process, KEGG, and WikiPathways. (**F**) GSEA highlighting pathways enriched in TIM3_high (positive NES) versus TIM3_low (negative NES). (**G**) Dot plot of curated marker genes comparing TIM3_high and TIM3_low groups. (**H**) Violin plots of functional module scores comparing TIM3_high and TIM3_low groups.

To formalize TIM3–linked myeloid states, we dichotomized tumors into TIM3_high and TIM3_low (TIM3_high n = 2258; TIM3_low n = 4450; Fig. 3A). This stratification uncovered a pronounced shift in TAM architecture: SPP1^+^ TAMs were selectively expanded in TIM3_high, whereas other myeloid subsets showed comparatively smaller or heterogeneous changes (Fig. 3B, *p* = 3.8 × 10^−^^4^), positioning the osteopontin-associated macrophage program as a dominant cellular correlate of elevated HAVCR2. The relative contributions of PCA, mHSPC, and CRPC differed between TIM3_high and TIM3_low groups, suggesting that TIM3–high macrophage states may be preferentially represented in advanced disease contexts (Fig. 3C). Differential expression analysis showed enrichment of HAVCR2 and co-regulated genes in TIM3_high cells, with reciprocal patterns in TIM3_low cells (Fig. 3D), supporting TIM3 stratification as a meaningful state definition and motivating downstream analyses in TIM3_high ecosystems.

Integrated functional analyses consistently indicated that TIM3_high macrophages exhibit a remodeling- and recruitment-oriented program. GSEA showed that TIM3_high cells were positively enriched for pathways related to vasculature/tissue repair, inflammatory responses, and cell migration and adhesion, whereas TIM3_low cells were preferentially enriched for housekeeping metabolic programs including ribosome biogenesis/protein translation and mitochondrial oxidative phosphorylation/aerobic respiration (Fig. 3E,F). At the gene level, TIM3_high macrophages showed elevated expression of inflammatory and chemokine-associated transcripts, accompanied by increased abundance of extracellular matrix and tissue-remodeling genes, including IL1B, TNF, IL6, CCL3, CCL4, CXCL9, CXCL10, CXCL11, and SPP1, FN1, THBS1, MMP9. In contrast, TIM3_low macrophages exhibited higher expression of antigen processing and presentation machinery, with particularly pronounced enrichment of MHC class II–associated genes, including HLA-DRA, HLA-DPA1, HLA-DPB1, CD74, and the MHC-II transcription activators CIITA (Fig. 3G). Building on the gene- and pathway-level differences between TIM3_high and TIM3_low macrophages, we next asked whether these transcriptional shifts translate into coherent functional modules at the state level. Consistent with a polarization split, TIM3_low macrophages exhibited a higher M1-like inflammatory signature (M1_1), whereas TIM3_high macrophages showed preferential enrichment of M2-like programs (M2_1) together with elevated lipid-associated macrophage (LAM_1) and ECM remodeling (ECM_1) modules (Fig. 3H). In parallel, chemokine-related activity (Chemokine_1) tended to be higher in the TIM3_high group, aligning with their “recruitment/remodeling” transcriptomic features, while antigen presentation (AP_1), interferon response (IFN_1), and phagocytosis-related (Phago_1) modules displayed relatively modest differences between groups. Taken together, these module-level patterns reinforce TIM3 not only to mark macrophage abundance, but also to depict a functional state transition, in which TIM3_high macrophages adopt a more M2-like tissue repair and ECM-related phenotype, while TIM3_low macrophages retain strong M1 inflammatory signatures, providing a mechanistic basis for subsequent analysis of upstream regulation and microenvironment enhancement TIM3_high niches. To test clinical generalizability, we derived a TIM3_Mph ssGSEA signature and evaluated it in independent bulk cohorts. TIM3_Mph scores increased with advancing stage in GSE116918 (Kruskal–Wallis, *p* = 0.045) and were reproducibly higher in more advanced stage groups in ICGC_PRAD_CA_seq (*p* = 0.018; Fig. S2A). In TCGA-PRAD, high TIM3_Mph stratified significantly worse PFI (log-rank *p* = 0.010), whereas OS separation was not significant (*p* = 0.539; Fig. S2B). This PFI-biased association is consistent with the long clinical course and post-progression treatment heterogeneity in prostate cancer, supporting TIM3_Mph as a microenvironmental marker more closely linked to progression/recurrence risk than to OS.

### 3.4 Regulon Activity Profiling Nominates PRDM1 as a Putative Upstream Regulator of the TIM3_High Macrophage State

**Figure 4:**
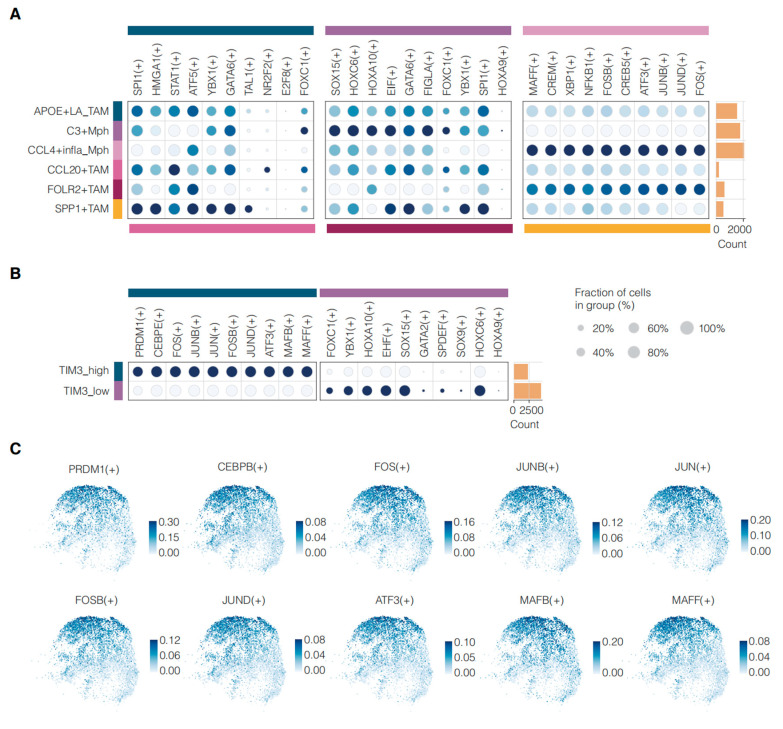
Regulon activity profiling nominates PR domain zinc finger protein 1 (PRDM1) as a putative upstream regulator of the TIM3_high macrophage state. (**A**) Heatmap/dot plot of regulon activities across TAM subclusters. (**B**) Differential regulon activity between TIM3_high and TIM3_low macrophages. (**C**) UMAP overlays of representative TF regulon activities. Regulon activity quantified using an AUCell-like scoring framework.

To move beyond differential gene expression and toward candidate regulators, we reconstructed gene regulatory networks and quantified TF regulon activity at single-cell resolution. Regulon landscapes across myeloid subclusters mirrored the underlying TAM-state architecture, revealing discrete, state-biased TF modules rather than uniform activation (Fig. 4A). Notably, comparison of TIM3_high versus TIM3_low macrophages identified PRDM1 regulon activation as a defining feature of the TIM3_high compartment, accompanied by elevated activity of CEBPB and AP-1/stress-response programs (including CEBPB, FOS/JUN family members, and ATF3) (Fig. 4B). In the low-dimensional embedding, these activity signals were spatially enriched within the TIM3_high niche, arguing that HAVCR2 upregulation is embedded within a broader, coordinated transcriptional program rather than reflecting diffuse or stochastic expression (Fig. 4C). PRDM1 (also known as BLIMP-1) is a lineage-instructive transcriptional regulator that enforces durable immune-state transitions by coordinating broad transcriptional and epigenetic programs [27]. Within the mononuclear phagocyte system, PRDM1 has been implicated in shaping macrophage differentiation and functional outputs in a context-dependent manner, positioning it as a plausible “state-locking” factor for clinically relevant myeloid phenotypes [28] Notably, recent evidence further supports a direct role for BLIMP-1 in macrophage polarization and metabolic reprogramming, reinforcing the concept that PRDM1 can couple upstream regulatory control to stable effector states [29]. These properties make PRDM1 a biologically compelling candidate to explain the emergence and persistence of the TIM3_high macrophage program observed in our atlas. Accordingly, we next interrogated whether PRDM1 tracks with TIM3 expression across cohorts and directly licenses TIM3 transcription.

### 3.5 PRDM1 Is Clinically Linked to HAVCR2/TIM3 and Directly Transactivates the HAVCR2 Promoter

**Figure 5:**
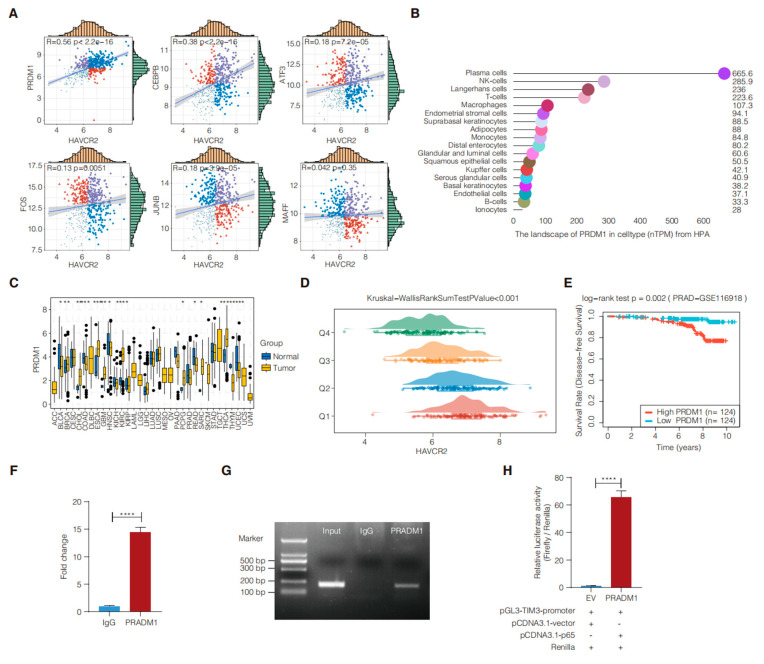
**PRDM1 correlates with HAVCR2, predicts adverse outcome, and directly activates the TIM3 promoter.** (**A**) Correlation of HAVCR2 with PRDM1 and candidate TFs (Pearson/Spearman as indicated): PRDM1 R = 0.56, *p* < 2.2 × 10^−^^16^. (**B**) Cell-type expression landscape of PRDM1 (nTPM) curated from the Human Protein Atlas (HPA), highlighting immune-lineage enrichment. (**C**) Pan-cancer comparison of PRDM1 expression between tumor and normal tissues across TCGA cancer types; statistical significance is annotated. (**D**) HAVCR2 expression across PRDM1 expression quartiles (Q1–Q4); group differences were assessed using the KM test (*p* < 0.001). (**E**) K-M analysis in GSE116918. (**F**) ChIP–qPCR enrichment relative to IgG control. (**G**) Representative ChIP-PCR gel electrophoresis showing Input, IgG, and PRDM1 immunoprecipitates. (**H**) Dual-luciferase reporter assay showing that PRDM1 overexpression increases TIM3 promoter activity. Firefly luciferase signals were normalized to Renilla luciferase. *p < 0.05; **p < 0.01; ***p < 0.001; ****p < 0.0001.

To validate the regulon-based inference in independent datasets, we interrogated bulk transcriptomes and observed a robust positive association between PRDM1 and HAVCR2 (R = 0.56, *p* < 2.2 × 10^−^^16^), consistent with PRDM1-aligned macrophage programs tracking with TIM3 expression. Other transcription factors (TFs) enriched under high TIM3 expression include CEBPB, ATF3, FOS, and JUNB, which show weak to moderate positive correlation, while MAFF and HAVCR2 demonstrate no significant association (Fig. 5A), supporting a coordinated inflammatory regulatory context rather than an isolated TF–target pair. According to Human Protein Atlas (HPA), PRDM1 expression is predominantly enriched in immune lineages, with the highest abundance observed in plasma cells, and appreciable expression also detected in NK cells, T cells, and monocytes/macrophages. This cell-type distribution is consistent with PRDM1 functioning as an immunoregulatory transcription factor (Fig. 5B). Consistently, TCGA pan-cancer analyses reveal tumor–normal differences in PRDM1 expression across multiple malignancies, including PRAD (Fig. 5C). Stratifying tumors by PRDM1 quartiles uncovered a stepwise increase in HAVCR2 levels, indicating a tiered relationship between PRDM1 abundance and TIM3 expression (Fig. 5D). Clinically, elevated PRDM1 was associated with shorter disease-free survival in GSE116918 (log-rank *p* = 0.002), linking this axis to aggressive disease behavior (Fig. 5E).

ChIP-qPCR showed a significant enrichment of PRDM1 immunoprecipitation over the IgG control at the targeted promoter region, with a marked increase in fold enrichment (*p* < 0.0001). This finding was further supported by ChIP–PCR, which yielded a specific amplicon in the PRDM1 pull-down, collectively indicating direct PRDM1 occupancy at the TIM3 promoter and providing molecular evidence for transcriptional licensing (Fig. 5F,G). In a pGL3–TIM3-promoter reporter assay, PRDM1 overexpression significantly increased normalized Firefly/Renilla luciferase activity relative to the empty vector (*p* < 0.001), demonstrating that PRDM1 not only binds the promoter but also functionally enhances TIM3 promoter-driven transcription (Fig. 5H). Given that TIM3 is frequently discussed in the context of lymphoid checkpoint biology, the PRDM1-dependent transactivation of HAVCR2 raised an immediate question: whether this axis in PCa primarily reflects a macrophage-intrinsic program or is embedded within broader checkpoint architectures involving T/NK compartments and intercellular crosstalk. Taken together, we provide a high-resolution annotation of the lymphoid compartment by re-clustering T/NK cells into naïve/central-memory populations, effector-like CD8 subsets, FOXP3^+^ regulatory T cells, γδT-related states, and an NK-like cytotoxic population supported by canonical marker expression (Fig. S3A). This refined lymphoid map offers an essential reference for interpreting checkpoint distribution and immune-state heterogeneity in PCa (Fig. S3B,C). Importantly, when integrated with our main analyses demonstrating that TIM3 is predominantly enriched within the monocyte–macrophage lineage rather than defining a major lymphoid-associated cluster, these results support the interpretation that the TIM3 axis in our study represents a myeloid-embedded functional state rather than a general feature of T-cell subsets. Accordingly, we prioritized downstream mechanistic interrogation centered on macrophage regulatory circuitry and microenvironmental reinforcement.

### 3.6 GALECTIN Signaling Points to an LGALS9–TIM3 Reinforcement Loop in TIM3_High Niches

**Figure 6:**
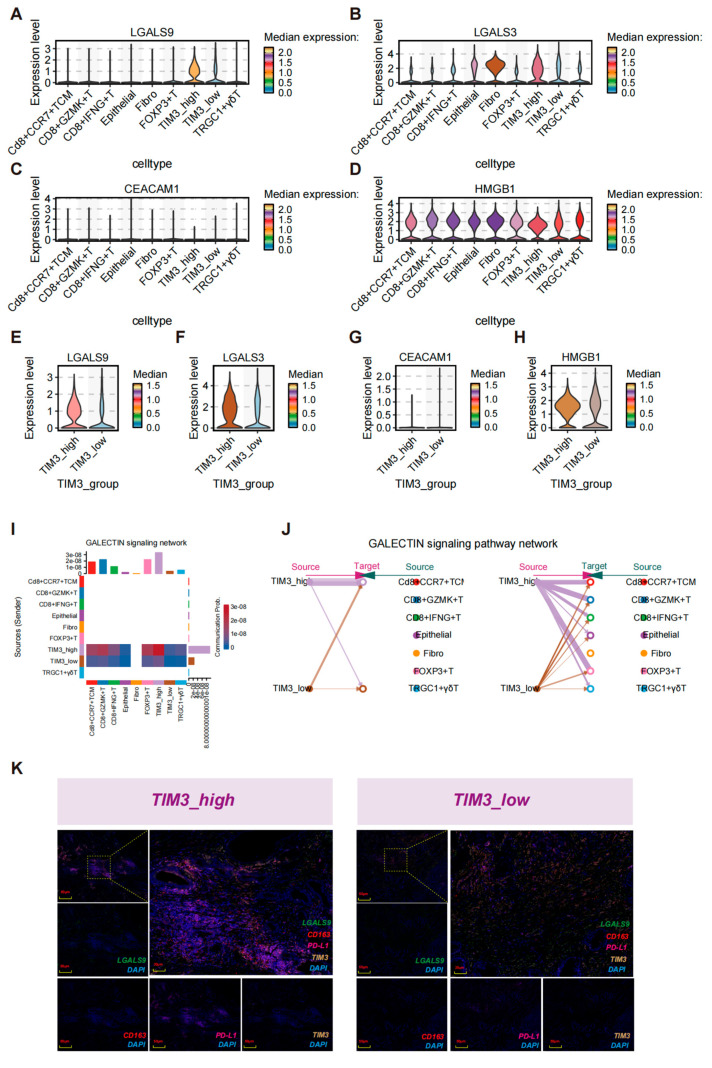
**Galectin signaling points to an LGALS9–TIM3 reinforcement loop in TIM3_high niches.** (**A–D**) Violin plots showing expression distributions of LGALS9 (**A**), LGALS3 (**B**), CEACAM1 (**C**) and HMGB1 (**D**) across annotated cell types. Median expression is indicated by the color scale. (**E–H**) Group-wise comparisons of the expression of LGALS9 (**E**), LGALS3 (**F**), CEACAM1 (**G**) and HMGB1 (**H**) between TIM3_high and TIM3_low cells. (**I**) Cell–cell communication inference for the GALECTIN signaling pathway. Heatmap depicts inferred communication probabilities between sender (source) and receiver (target) cell types. (**J**) Network visualizations summarizing GALECTIN pathway interactions involving TIM3_high and TIM3_low states, highlighting predominant sources and target patterns. (**K**) Multiplex immunofluorescence validation of LGALS9–TIM3 co-localization in TIM3_high tumors.

To probe ligand-driven reinforcement of TIM3_high myeloid niches, we prioritized the best-characterized TIM3 ligands that represent distinct extracellular input classes and have established immunoregulatory consequences. Galectin-9 (LGALS9) is the canonical lectin ligand most frequently linked to TIM3-dependent inhibitory signaling, whereas CEACAM1 acts as a heterophilic binding partner required for TIM3–mediated T-cell inhibition and tolerance. HMGB1, an alarmin released under stress or tissue damage, connects TIM3 to suppression of nucleic-acid-driven innate immune activation in tumor-associated antigen-presenting cells [30]. We additionally profiled LGALS3 to capture the broader galectin-rich milieu, which is frequently abundant across tumor, immune, and stromal compartments, and to determine whether a GALECTIN-centered microenvironment co-segregates with TIM3_high ecosystems (Fig. 6A–D) [31]. Compared with TIM3_low cells, TIM3_high cells displayed elevated LGALS9 and a modest increase in LGALS3, consistent with enhanced GALECTIN signaling and nominating the LGALS9-TIM3 axis as a candidate reinforcement pathway (Fig. 6E,F). In contrast, CEACAM1 was largely undetectable and showed no meaningful group difference, arguing against a major contribution of the CEACAM1-linked TIM3 route in this dataset. HMGB1 was ubiquitous with only minor between-group variation, consistent with a nonspecific stress/injury-associated background signal rather than a TIM3_high-selective feature (Fig. 6G,H).

To move from expression patterns to functional signaling, we applied CellChat to model ligand–receptor communication between TIM3_high/low macrophages and other cell populations. GALECTIN signaling emerged as a major pathway associated with the TIM3_high niche (Fig. 6I). At single interaction resolution, LGALS9–HAVCR2 was among the top-ranked ligand–receptor pairs preferentially active in TIM3_high networks (Fig. 6J). To examine GALECTIN-related features in TIM3_high tumors at the tissue level, multiplex immunofluorescence staining for LGALS9, TIM3, CD163, PD-L1, and DAPI was performed in TIM3_high and TIM3_low prostate cancer samples (Fig. 6K). In TIM3_high tumors, LGALS9 expression was clearly increased and mainly observed in regions enriched for CD163^+^ myeloid cells, where it showed evident spatial overlap with TIM3 and appeared as focal clusters. By contrast, TIM3_low tumors displayed weaker LGALS9 and TIM3 signals with little co-localization in CD163^+^ cells. In addition, LGALS9^+^TIM3^+^CD163^+^ cells in TIM3_high regions often expressed PD-L1, consistent with the LGALS9–TIM3 signaling patterns inferred from single-cell and CellChat analyses. Combined with PRDM1-associated transcriptional regulation of HAVCR2, these results support a two-layer model in which endogenous induction of TIM3 is enhanced by extrinsic LGALS9 binding to form a self-sustaining macrophage TIM3_high. This framework has led to a broader exploration of co-enrichment propagation procedures, particularly chemotaxis and adhesion axes, which enable the integration of TIM3 biology with macrophage localization and matrix remodeling.

### 3.7 Communication Rewiring in TIM3_High Niches Prioritizes MIF–CXCR4 Chemotaxis and FN1-Integrin Adhesion Axes

**Figure 7:**
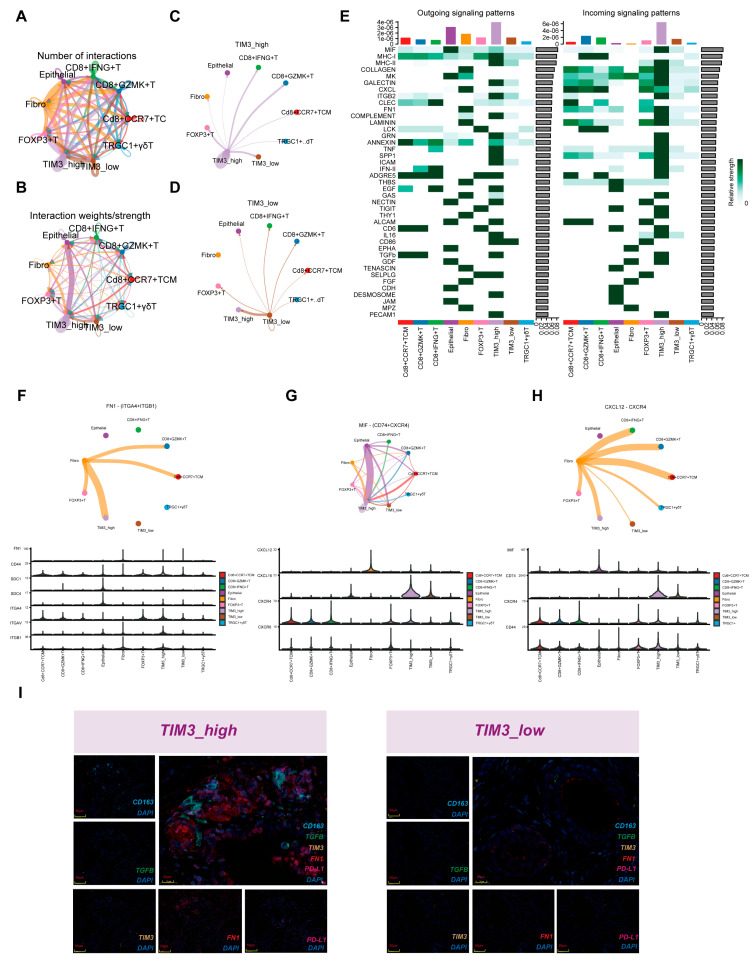
**Communication rewiring in TIM3_high niches prioritizes MIF–CXCR4 chemotaxis and FN1–integrin adhesion axes.** (**A**) Circle plots depicting the number of predicted ligand–receptor interactions within the inferred intercellular communication network across annotated cell types. (**B**) Circle plots depicting the interaction weights/strength of predicted ligand–receptor interactions within the inferred intercellular communication network across annotated cell types. (**C**) Signaling role network plots centered on TIM3_high. (**D**) Signaling role network plots centered on TIM3_low. (**E**) Heatmaps of outgoing and incoming signaling patterns across major pathways inferred by CellChat, highlighting differential engagement of chemotactic, ECM/adhesion, and immune-regulatory programs. (**F**) Chord diagrams depicting FN1-(ITGA4+ITGB1) axes. (**G**) Chord diagrams depicting MIF-(CD74+CXCR4) axes. (**H**) Chord diagrams depicting MIF-(CD74+CXCR4) axes. Ridge plots below each chord diagram show expression distributions of the corresponding ligand and receptor components across cell types, providing transcriptional support for inferred interactions. (**I**) Representative multiplex immunofluorescence images of TIM3_high and TIM3_low tumors stained for CD163, TIM3, FN1, TGFβ, PD-L1, and DAPI. Enlarged views highlight the spatial co-localization of CD163^+^ myeloid cells with TIM3 and immunosuppressive ligands in TIM3_high regions, compared with sparse and weakly overlapping signals in TIM3_low tumors. Scale bars are indicated.

We next performed a systems-level comparison of intercellular communication centered on TIM3_high versus TIM3_low macrophages using CellChat, quantifying both interaction number and aggregate signaling strength across major cell populations. TIM3_high networks showed a global gain in communication together with a marked redistribution of pathway usage toward recruitment, positioning, and matrix-engagement programs (Fig. 7A–E). Based on these inferences, we compared the expression of key ligand–receptor components and functionally relevant genes between the TIM3_high and TIM3_low groups to verify that the predicted communication programs are supported at the transcriptional level; notably, ITGB1 and CXCR4 showed significant differences between the two states (Fig. S4A).

Pathway-level decomposition and ligand–receptor ranking converged on three tightly coupled axes. First, FN1–integrin signaling—notably FN1 (ITGA4/ITGB1)—was strengthened, aligning with adhesion-dependent retention and extracellular matrix coupling (Fig. 7F). Second, MIF signaling was enhanced, with MIF (CD74/CXCR4) emerging as a dominant interaction module, consistent with a microenvironment permissive for myeloid persistence and motility (Fig. 7G). Third, CXCL12–CXCR4 communication was increased, implicating chemokine-driven spatial organization of immune cells within the TIM3_high niche (Fig. 7H). Multiplex immunofluorescence analysis revealed distinct spatial immune microenvironment features between TIM3_high and TIM3_low tumors (Fig. 7I). In TIM3_high regions, CD163^+^ myeloid cells were densely distributed and showed pronounced co-localization with TIM3, FN1, TGFβ, and PD-L1, forming clustered immunosuppressive niches. In contrast, TIM3_low tumors exhibited reduced CD163^+^ cell infiltration and markedly weaker expression of TIM3-associated ligands, with limited spatial overlap among these markers. Collectively, the inferred communication landscape provides orthogonal support for the chemokine- and ECM-remodeling programs transcriptionally enriched in TIM3_high macrophages and highlights potentially targetable signaling dependencies.

### 3.8 Recombinant LGALS9 Induces TIM3-Dependent M2-Like Polarization in THP-1-Derived Macrophage-Like Cells and Is Attenuated by TIM3 Blockade

**Figure 8:**
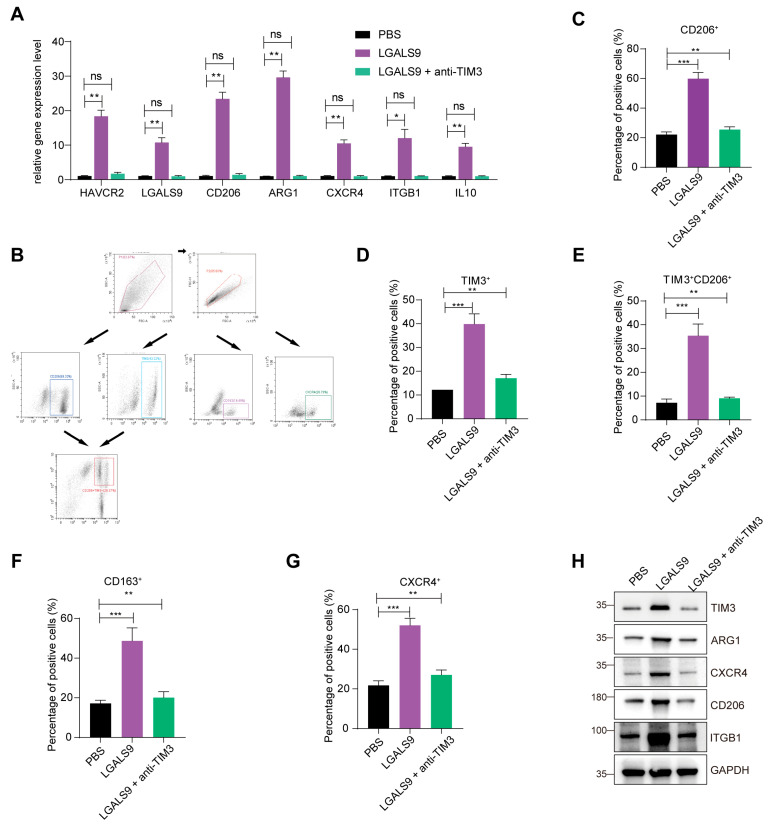
Galectin-9 (LGALS9) drives TIM3–dependent M2-like polarization in the human acute monocytic leukemia cell line (THP-1) macrophage-like cells and is reversible with TIM3 blockade. (**A**) Reverse Transcription quantitative PCR (RT-qPCR) analysis of HAVCR2 (TIM3), LGALS9, CD206 (MRC1), ARG1, CXCR4, ITGB1, and IL10 under PBS/control, LGALS9, and LGALS9 + anti-TIM3 conditions. Data are shown as relative expression normalized to GAPDH and control group. (**B**) Representative flow cytometric gating strategy for the identification and hierarchical selection of target cell populations. (**C**–**G**) Statistical quantification of flow cytometry data showing the percentage of (**C**) CD206^+^, (**D**) TIM3^+^, (**E**) TIM3^+^CD206^+^, (**F**) CD163^+^, and (**G**) CXCR4^+^ cells in each group. (**H**) WB analysis of TIM3, ARG1, CD206, CXCR4, and ITGB1 with GAPDH as a loading control under the same conditions. *p < 0.05, **p < 0.01, ***p < 0.001, ns, no significance.

In a THP-1–derived macrophage-like model, LGALS9 stimulation induced a coordinated TIM3–linked polarization program. At the transcriptional level, LGALS9 increased TIM3 together with M2-like markers (MRC1/CD206, ARG1, IL10) and concomitantly elevated chemokine/adhesion-associated transcripts (CXCR4, ITGB1). Neutralizing anti-TIM3 markedly dampened these LGALS9-driven increases, returning expression toward baseline, consistent with a TIM3–dependent response (Fig. 8A). Phenotypically, flow cytometry corroborated these findings: LGALS9 increased the proportions of CD206^+^, TIM3^+^, and TIM3^+^CD206^+^ cells, while anti-TIM3 co-treatment significantly reduced each readout, thereby coupling TIM3 induction with an M2-like surface phenotype within the same cellular state. LGALS9 also increased CD163^+^ and CXCR4^+^ fractions, and both effects were attenuated by TIM3 blockade, suggesting that the LGALS9–TIM3 axis extends beyond polarization markers to reinforce recruitment/localization-associated features (Fig. 8B–G). Consistently, immunoblotting showed increased protein abundance of TIM3, ARG1, CD206, CXCR4 and ITGB1 following LGALS9 exposure, which was broadly diminished by anti-TIM3 co-treatment (Fig. 8H). Collectively, these results support LGALS9–TIM3 as an experimentally tractable reinforcement circuit that functionally sustains an M2-like, chemotaxis/adhesion-leaning program, providing orthogonal validation for the communication-based inference described above.

## 4 Discussion

TIM3, a well-characterized immune checkpoint, has garnered significant attention for its role in regulating both T-cell and myeloid immune functions. While its involvement in T-cell exhaustion has been well-documented in various malignancies [32,33], its role within the myeloid compartment, particularly in PCa, has remained underexplored. Recent evidence highlights myeloid cells as key drivers of the immunosuppressive tumor microenvironment (TME) in PCa [34]. Unpacking their heterogeneity could therefore explain how this suppressive niche forms. Given TIM3’s diverse functional repertoire and its emerging relevance across immune lineages, understanding its full range of actions in PCa becomes crucial.

In this study, we reveal several key findings regarding the role of TIM3 in PCa. First, we show that TIM3 is predominantly expressed in the myeloid compartment, particularly in macrophages, rather than in T cells, highlighting its myeloid-centered role in PCA. Second, TIM3 expression levels correlate with patient prognosis, with higher TIM3 expression associated with poor PFS in multiple clinical cohorts, suggesting its potential as a progression marker. Lastly, we identify PRDM1 as a key upstream regulator of TIM3 expression, demonstrating that TIM3 is transcriptionally activated by PRDM1, and we uncover ligand-driven reinforcement mechanisms, particularly through the LGALS9–TIM3 axis, that sustain TIM3 high macrophage phenotypes. These findings support the notion that TIM3 functions as a central regulatory node in PCa, influencing immune microenvironment remodeling, macrophage polarization, and disease progression.

Mechanistically, our data implicate PRDM1 as a proximal transcriptional driver that “licenses” the TIM3_high program. PRDM1 has established roles in immune-state specification and durable transcriptional transitions across immune lineages, and in mononuclear phagocytes it can constrain activation trajectories and reprogram functional outputs in a context-dependent manner [27,28,35]. Consistent with these principles, we identify PRDM1 as an upstream regulator correlated with TIM3 and demonstrate direct promoter engagement and transcriptional activation, supporting a state-locking model in which PRDM1 provides intrinsic stability to TIM3 expression and couples it to broader stress–response regulatory architectures [29]. This framing has practical implications: transcriptional licensing implies that TIM3–high macrophage states may persist even under fluctuating microenvironmental cues, thereby sustaining immunosuppressive niches unless upstream regulatory logic is disrupted [36].

At the extrinsic layer, ligand mapping and functional perturbation converge on LGALS9–TIM3 as a candidate reinforcement circuit that amplifies TIM3–linked polarization programs. TIM3 binds multiple ligands, among which galectin-9 has been extensively studied as a potent immunoregulatory partner in cancer and chronic inflammation [37,38]. Another study demonstrates that Galectin-9 promotes colon cancer progression by inducing macrophage polarization toward the M2 phenotype, which contributes to tumor growth and immune suppression [39]. We have experimentally validated the results to induce LGALS9 stimulation to induce upregulation of TIM-3 expression, enhance M2-like polarization, and promote chemotaxis and adhesion-related genes. Importantly, neutralizing TIM-3 mitigated these effects, confirming the ligand-driven reinforcement predicted by our cell-to-cell communication assay. These findings support a two-step model where PRDM1 authorizes TIM-3 expression at the transcriptional level and LGALS9 binds to maintain a polarization program, thereby promoting the persistence of the TIM3 high macrophage niche.

Our cell–cell communication analysis identifies TIM3-high macrophages within a niche conducive to recruitment and stromal anchoring, driven by strengthened MIF–(CD74/CXCR4), CXCL12–CXCR4, and FN1–integrin interactions. Notably, MIF and CXCL12 signaling have been implicated in immune cell trafficking, tumor vascularity, and metastatic niche formation, suggesting targetable dependencies that could cooperate with TIM3 to sustain immunosuppressive macrophage niches [40,41,42]. These axes therefore nominate targetable signaling dependencies that may cooperate with TIM-3 in sustaining immunosuppressive, remodeling-prone macrophage ecosystems. Our findings carry potential clinical implications for both diagnosis and treatment of prostate cancer. The myeloid TIM3 signature (TIM3_Mph) demonstrated independent prognostic value for progression-free interval across multiple cohorts, suggesting its utility as a microenvironmental biomarker to complement existing genomic stratifiers such as mismatch repair deficiency and CDK12 mutation status in guiding patient selection for immune-based interventions [43].

From a therapeutic perspective, several anti-TIM3 monoclonal antibodies are currently under clinical investigation, and the PRDM1-licensed TIM3-high macrophage program identified here provides a biological rationale for evaluating these agents in prostate cancer [44,45]. Co-targeting the LGALS9–TIM3 reinforcement loop or combining TIM3 blockade with CXCR4 antagonists may offer synergistic benefits by simultaneously disrupting immunosuppressive macrophage polarization and tumor-promoting niche formation, warranting future preclinical and clinical investigation.

Although TIM3 is well-established as an inhibitory receptor on exhausted T cells, HAVCR2 expression in our atlas was relatively enriched in the monocyte/macrophage lineage compared with T/NK cells, consistent with the immune-cold nature of prostate cancer. Technical limitations of scRNA-seq, such as transcript dropout, may also partially contribute to the low HAVCR2 signal in T/NK cells [7,46]. These observations suggest that TIM3 biology in prostate cancer is predominantly myeloid-embedded, representing a functionally distinct program from its canonical role in T-cell exhaustion.

Several limitations warrant emphasis. First, the single-cell atlas integrates public datasets with heterogeneous sampling and processing, and residual confounding cannot be fully excluded despite batch-aware strategies [47,48]. Second, ligand–receptor inference is probabilistic and transcript-based; it cannot establish directionality or protein-level engagement, and thus remains hypothesis-generating without orthogonal proteomic validation [49,50]. Third, outcome stratification based on cutpointing may introduce optimism bias if not prospectively validated, and should be complemented by continuous-effect models and independent prospective cohorts [51]. In addition, THP-1–derived macrophages are a tractable system for perturbation experiments but may not fully capture the heterogeneity and tissue-imprinted states of primary tumor-associated macrophages *in vivo*. Nonetheless, the PRDM1–TIM3 program and LGALS9–TIM3 reinforcement observed in THP-1 cells are concordant with our patient single-cell/spatial data, supporting biological relevance while warranting validation in primary TAMs.

## 5 Conclusion

In conclusion, our study reveals the pivotal role of the TIM3 myeloid axis in PCa by integrating single-cell spatial multi-omics, mIF and targeted experimental manipulations. We demonstrate that TIM3 is predominantly expressed in the monocyte/macrophage lineage, marking a state-specific macrophage program associated with chemokine signaling and ECM remodeling. PRDM1 directly transactivates HAVCR2, providing molecular evidence for TIM3 transcriptional licensing. Additionally, the LGALS9–TIM3 axis acts as a reinforcing loop, enhancing M2-like polarization and chemotaxis. Therefore, targeting the TIM3^+^ macrophage, especially in combination with macrophage recruitment/retention disruption, could overcome resistance and improve clinical outcomes.

## Data Availability

The raw data underlying this study were generated and deposited in the relevant repositories. Additional information can be obtained from the corresponding authors upon reasonable request.

## Supplementary Materials

The supplementary material is available online at https://www.techscience.com/doi/10.32604/or.2026.079316/s1.
