# Supplementary material for: PRDM1 Drives a TIM3+ Macrophage Immunosuppressive Niche via LGALS9 Signaling in Prostate Cancer Progression
Source: Oncol Res. 2026 Jun 16;34(7):26. doi: 10.32604/or.2026.079316 (PMC13292046; doi:10.32604/or.2026.079316)
Supplement: Supplementary file 1 [file OncolRes-34-79316-s001.zip › TSP_OR_79316-s001.docx]

**Table S1:Patients for multiplex immunofluorescence (mIF)**

| **Case** | **Inpatient No.** | **Sex** | **Age, year** | **Diagnosis** | **Prostate volume,** **cm** ×**cm**×**cm** | **Clinical stage** | **Gleason score** | **Initial PSA** |
| --- | --- | --- | --- | --- | --- | --- | --- | --- |
| Case1 | 17695377 | Male | 73 | Prostate cancer | 3×4×5 | T2cN0M0 | 4+3=7 | 71 |
| Case2 | 17628722 | Male | 73 | Prostate cancer | 3×4×5 | T2aN0M0 | 4+4=8 | 88 |
| Case3 | 17690882 | Male | 80 | Prostate cancer | 4×5×6 | T2bN0M0 | 3+4=7 | 35 |
| Case4 | 17700507 | Male | 72 | Prostate cancer | 3×4×5 | T2cN0M0 | 4+5=9 | 15 |
| Case5 | 17688550 | Male | 63 | Prostate cancer | 3×4×5 | T2aN0M0 | 3+3=6 | 7.3 |
| Case6 | 17700455 | Male | 73 | Prostate cancer | 3×4×5 | T2bN0M0 | 3+3=6 | 35 |
| Case7 | 17714604 | Male | 71 | Prostate cancer | 3×4×5 | T2bN0M0 | 4+4=8 | 20 |
| Case8 | 17717113 | Male | 75 | Prostate cancer | 4×5×5 | T2aN0M0 | 4+3=7 | 10.7 |
| Case9 | 17752687 | Male | 68 | Prostate cancer | 3×4×5 | T2cN0M0 | 5+4=9 | 22 |
| Case10 | 17755229 | Male | 70 | Prostate cancer | 3×4×5 | T2cN0M0 | 3+4=7 | 48 |

**Table S2: Primers used for Reverse Transcription quantitative PCR (RT-qPCR)**

| **Gene** | **Direction** | **Sequence 5’-3’** |
| --- | --- | --- |
| **HAVCR2 (TIM3)** | **Forward** | **CTGCTGCTACTACTTACAAGGTC** |
|  | **Reverse** | **GCAGGGCAGATAGGCATTCT** |
| **LGALS9** | **Forward** | **TTACTGGACCAATCCAAGGAGG** |
|  | **Reverse** | **AGCTGTTCTGAAAGTTCACCAC** |
| **MRC1 (CD206)** | **Forward** | **TCCGGGTGCTGTTCTCCTA** |
|  | **Reverse** | **CCAGTCTGTTTTTGATGGCACT** |
| **ARG1** | **Forward** | **GTGGAAACTTGCATGGACAAC** |
|  | **Reverse** | **AATCCTGGCACATCGGGAATC** |
| **CXCR4** | **Forward** | **ACTACACCGAGGAAATGGGCT** |
|  | **Reverse** | **CCCACAATGCCAGTTAAGAAGA** |
| **ITGB1** | **Forward** | **CCTACTTCTGCACGATGTGATG** |
|  | **Reverse** | **CCTTTGCTACGGTTGGTTACATT** |
| **IL10** | **Forward** | **GACTTTAAGGGTTACCTGGGTTG** |
|  | **Reverse** | **TCACATGCGCCTTGATGTCTG** |
| **GAPDH** | **Forward** | **GGAGCGAGATCCCTCCAAAAT** |
|  | **Reverse** | **GGCTGTTGTCATACTTCTCATGG** |


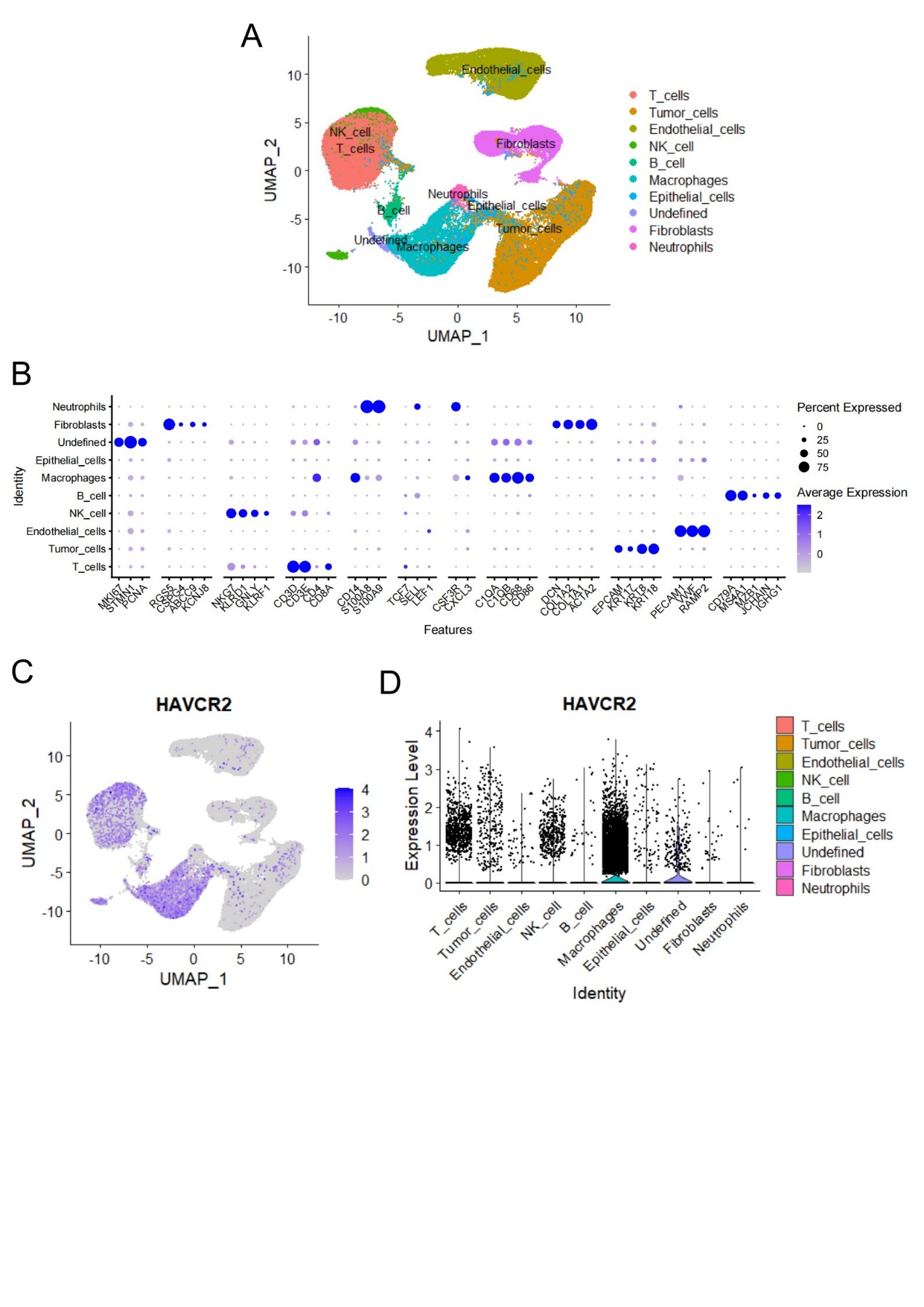


**Figure S1. Independent validation of myeloid-dominant TIM3 (HAVCR2) expression in an external single-cell RNA-seq cohort. (A)** Uniform Manifold Approximation and Projection (UMAP) visualization of major cell lineages identified in the independent single-cell RNA-seq dataset (GSA accession: HRA000823), including T cells, tumor cells, endothelial cells, Natural Killer cells (NK cells), B cells, macrophages, epithelial cells, fibroblasts, and neutrophils. Each cluster is colored by annotated cell type. **(B)** Dot plot showing the expression of canonical marker genes across annotated cell populations. Dot size represents the percentage of expressing cells; color intensity reflects average expression level. **(C)** UMAP feature plot displaying HAVCR2 expression across all cells, with color scale indicating expression level (0–4). **(D)** Violin plot showing HAVCR2 expression levels across all annotated cell populations, confirming relative enrichment in the macrophage compartment compared with T/NK cells and other lineages.

**
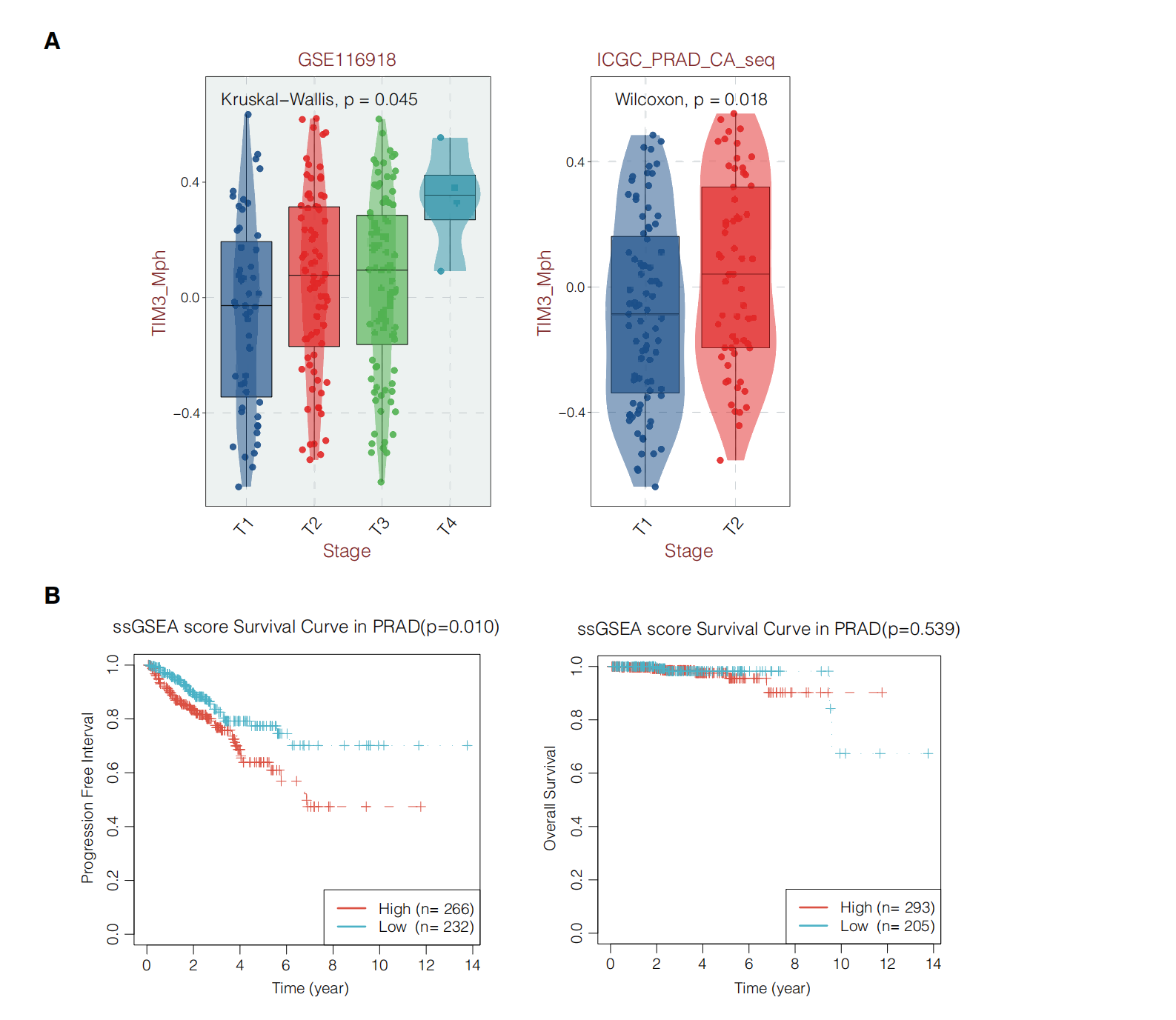
**

**Figure S2**. **TIM3 (HAVCR2)** **prognostic value across RNA Bulk Sequences.** **(A)** TIM3_Mph scores across T stages (GSE116918, Kruskal–Wallis *P* = 0.045, ICGC_PRAD, Wilcoxon *P*= 0.018). **(B)** Kaplan-Meier (K-M) plots demonstrated distinct overall survival (OS) and progression-free survival (PFS) outcomes for groups classified by TIM3-Mph.


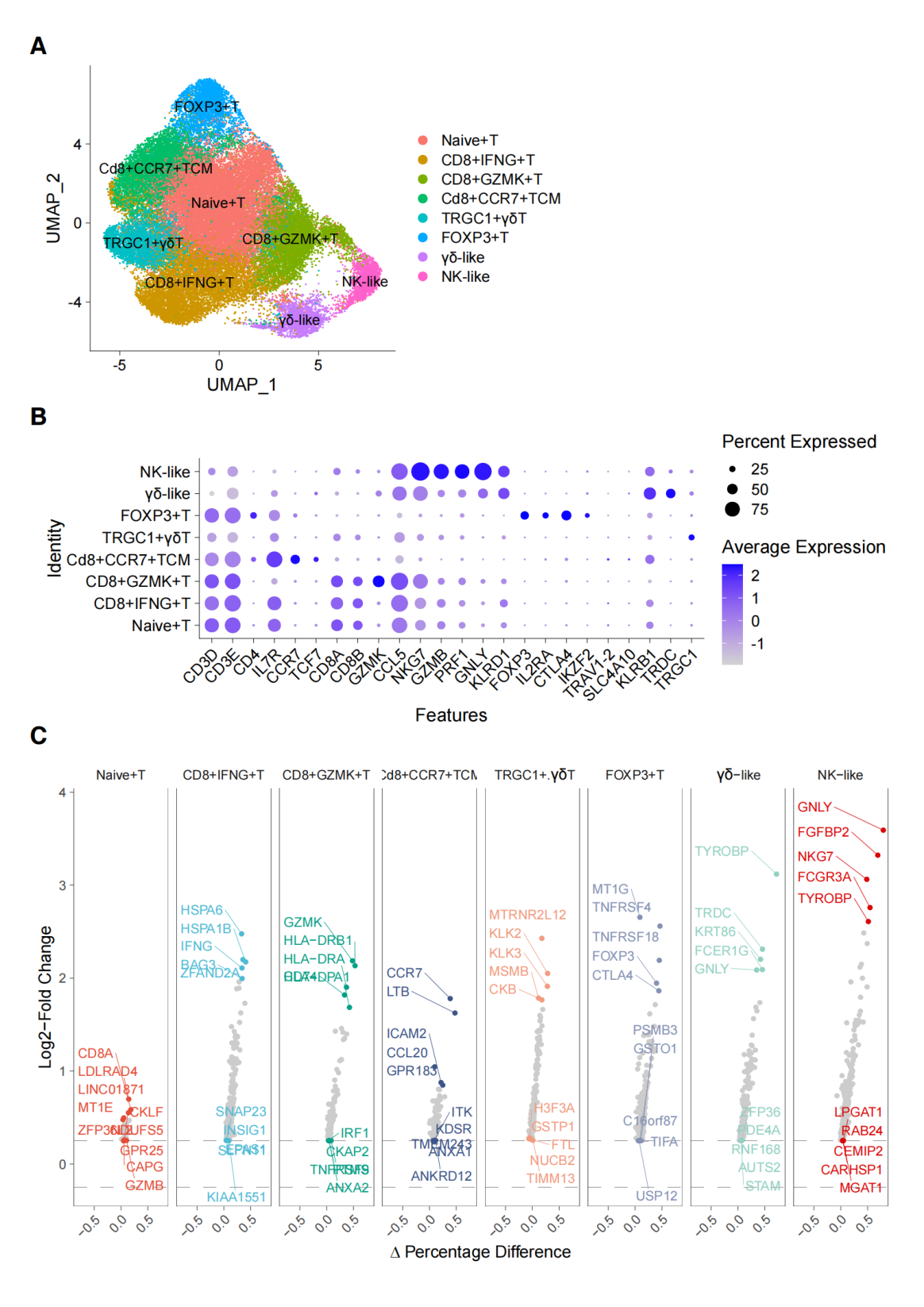


**Figure S3. T/NK compartment analysis (A)** UMAP visualization of re-clustered T/NK cells colored by annotated subsets, including Naive+T, CD8+IFNG+T, CD8+GZMK+T, CD8+CCR7+TCM, TRGC1+γδT, FOXP3+T, γδ-like, and NK-like populations. **(B)** Dot plot of canonical marker genes used for subset annotation. Dot size indicates the fraction of cells expressing each gene, and color represents scaled average expression. **(C)** Differential expression summary for each subset relative to the remaining T/NK cells.


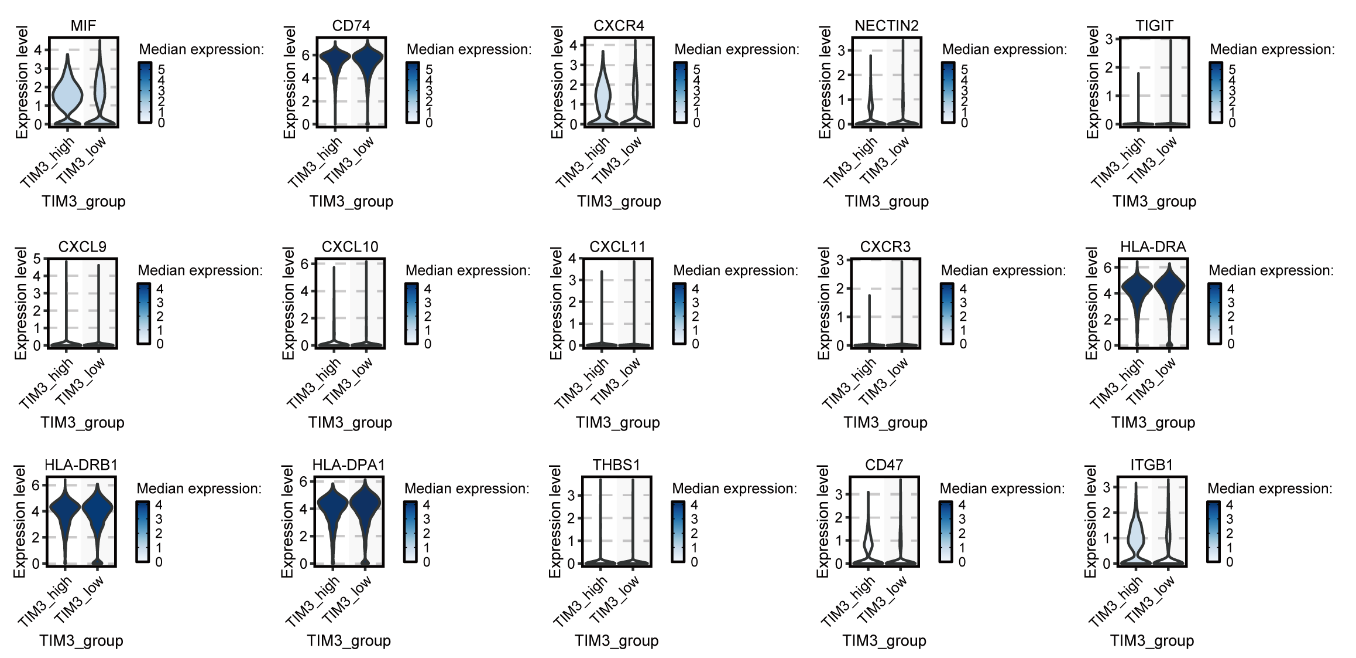


**Figure S4 Expression controls of key ligands/receptors and functionally relevant genes.** Violin plots comparing expression distributions of selected ligand/receptor components and functional markers between TIM3_high and TIM3_low cells, including MIF, CD74, CXCR4, NECTIN2, TIGIT, CXCL9/10/11, CXCR3, HLA-DRA/DRB1/DPA1, THBS1, CD47, and ITGB1.
