# Supplementary figures and images for: PRDM1 Drives a TIM3+ Macrophage Immunosuppressive Niche via LGALS9 Signaling in Prostate Cancer Progression
Source: Oncol Res. 2026 Jun 16;34(7):26. doi: 10.32604/or.2026.079316 (PMC13292046; doi:10.32604/or.2026.079316)

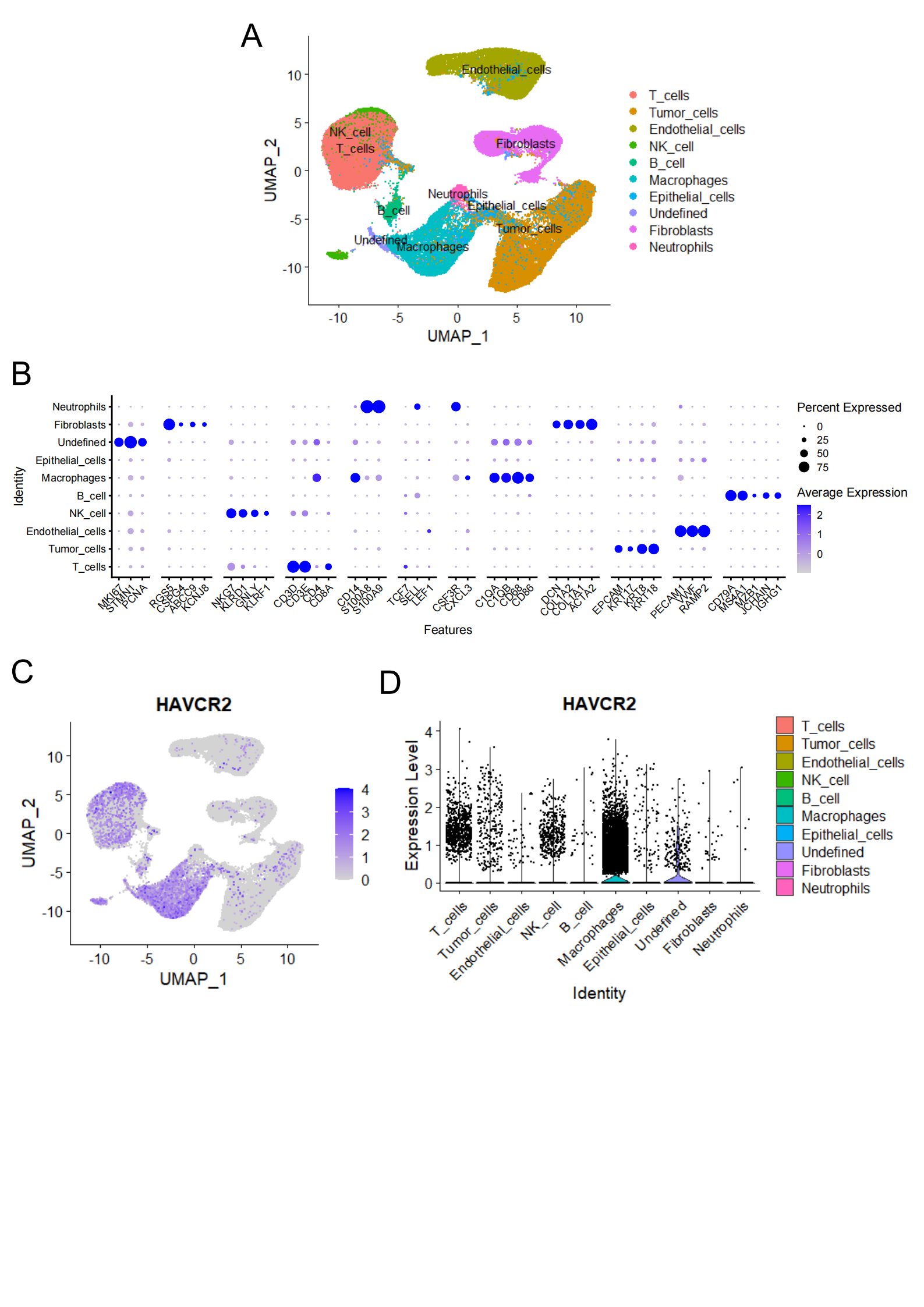

Supplement: Supplementary file 1 [file OncolRes-34-79316-s001.zip › Figure_S1.tif]

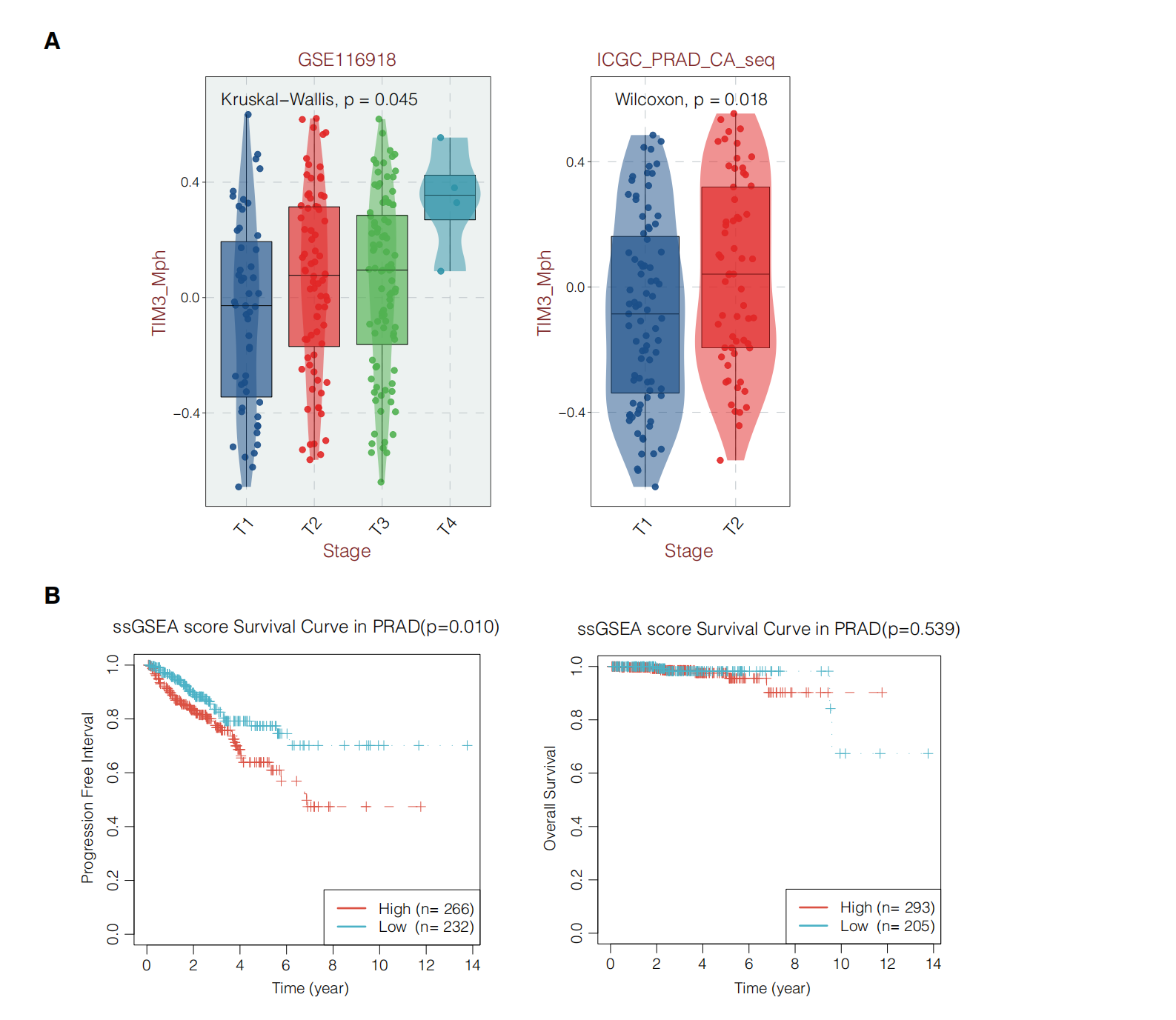

Supplement: Supplementary file 1 [file OncolRes-34-79316-s001.zip › Figure_S2.tif]

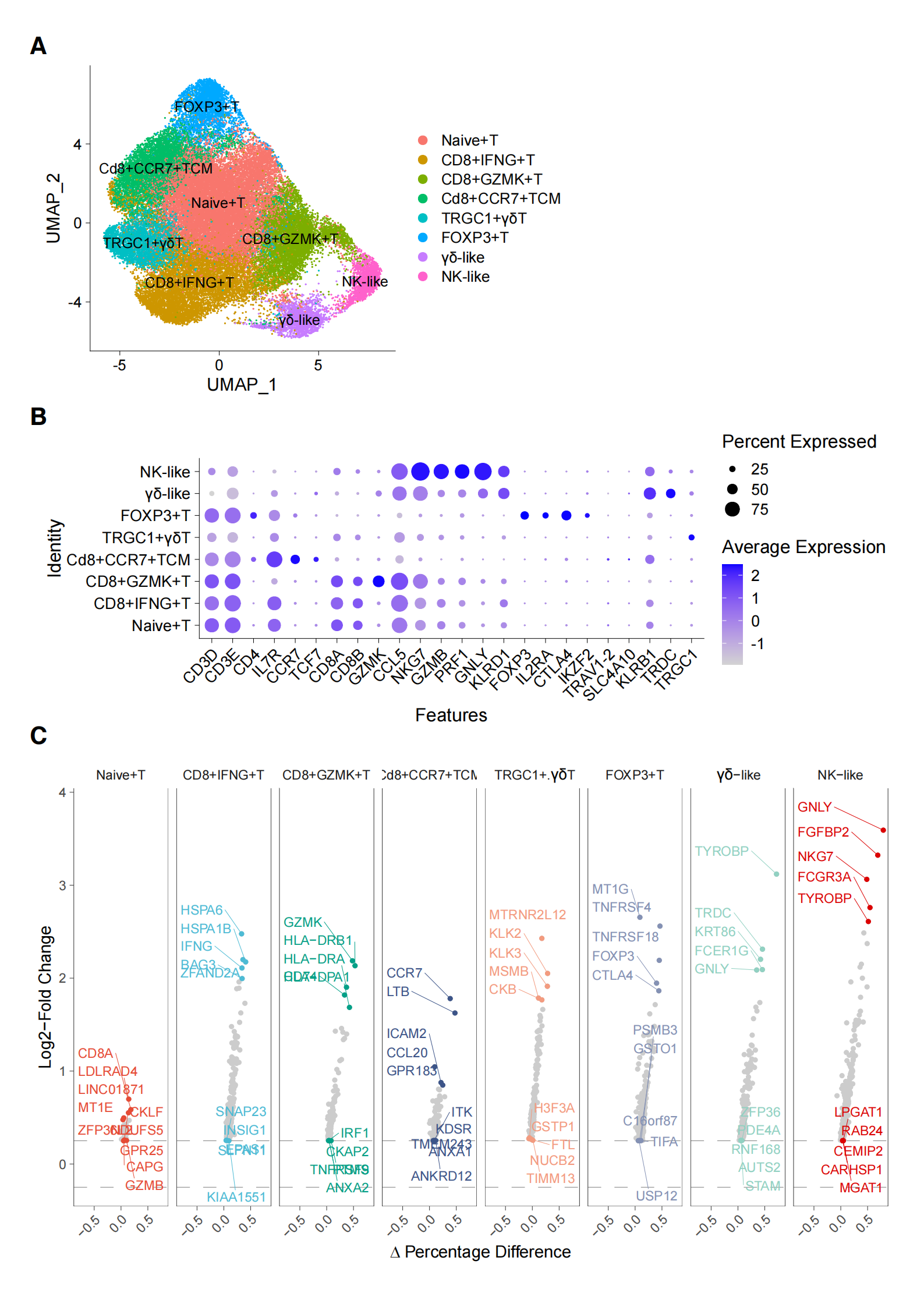

Supplement: Supplementary file 1 [file OncolRes-34-79316-s001.zip › Figure_S3.tif]

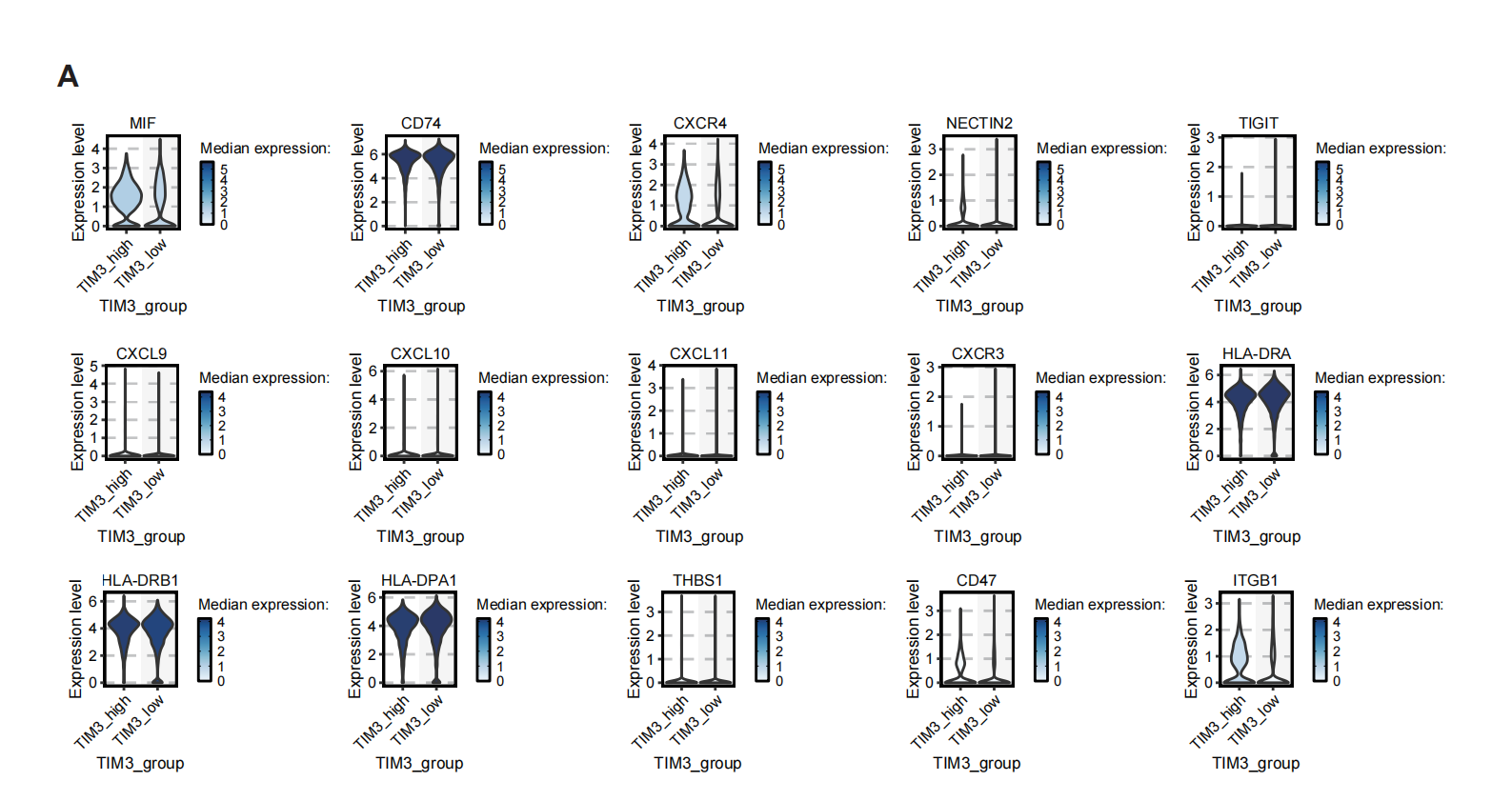

Supplement: Supplementary file 1 [file OncolRes-34-79316-s001.zip › Figure_S4.tif]
